# The Eneolithic cemetery at Khvalynsk on the Volga River

**DOI:** 10.1515/pz-2022-2034

**Published:** 2022-03-23

**Authors:** David W. Anthony, A.A. Khokhlov, S. A. Agapov, D. S. Agapov, R. Schulting, I. Olalde, D. Reich

**Affiliations:** Hartwick College, *emeritus*; & Harvard University, Human Evolutionary Biology, Cambridge, MA; Samara State University of Social Sciences and Education; Ecological and Cultural Association “Povolzje”, Samara; Historical, Ecological and Cultural Association “Povolzje”, Samara; University of Oxford, Scientific and Prehistoric Archaeology; Harvard Medical School Department of Genetics, Boston, MA; & CSIC–Universitat Pompeu Fabra, Institute of Evolutionary Biology, Barcelona; Harvard Medical School Department of Genetics; & Harvard University, Human Evolutionary Biology Department, Cambridge, MA; Howard Hughes Medical Institute, Boston, MA; Broad Institute of MIT and Harvard, Cambridge, MA

**Keywords:** Eneolithic, Russian steppe, mortuary archaeology, ritual sacrifice, copper metallurgy, ancient DNA, social differentiation

## Abstract

The genetically attested migrations of the third millennium BC have made the origins and nature of the Yamnaya culture a question of broad relevance across northern Eurasia. But none of the key archaeological sites most important for understanding the evolution of Yamnaya culture is published in western languages. These key sites include the fifth-millennium BC Khvalynsk cemetery in the middle Volga steppes. When the first part of the Eneolithic cemetery (Khvalynsk I) was discovered in 1977–79, the graves displayed many material and ritual traits that were quickly recognized as similar and probably ancestral to Yamnaya customs, but without the Yamnaya kurgans. With the discovery of a second burial plot (Khvalynsk II) 120m to the south in 1987–88, Khvalynsk became the largest excavated Eneolithic cemetery in the Don-Volga-Ural steppes (201 recorded graves), dated about 4500–4300 BCE. It has the largest copper assemblage of the fifth millennium BC in the steppes (373 objects) and the largest assemblage of sacrificed domesticated animals (at least 106 sheep-goat, 29 cattle, and 16 horses); and it produced four polished stone maces from well-documented grave contexts. The human skeletons have been sampled extensively for ancient DNA, the basis for an analysis of family relationships. This report compiles information from the relevant Russian-language publications and from the archaeologists who excavated the site, two of whom are co-authors, about the history of excavations, radiocarbon dates, copper finds, domesticated animal sacrifices, polished stone maces, genetic and skeletal studies, and relationships with other steppe cultures as well as agricultural cultures of the North Caucasus (Svobodnoe-Meshoko) and southeastern Europe (Varna and Cucuteni-Tripol’ye B1). Khvalynsk is described as a coalescent culture, integrating and combining northern and southern elements, a hybrid that can be recognized genetically, in cranio-facial types, in exchanged artifacts, and in social segments within the cemetery. Stone maces symbolized the unification and integration of socially defined segments at Khvalynsk.

## Introduction: the Yamnaya phenomenon and its origins

Recent large-scale studies of ancient DNA (aDNA) show that the Bronze Age populations in the steppes of present-day Russia and Ukraine migrated across both Europe and Asia during the Early Bronze Age (EBA) and the Middle Bronze Age (MBA) in the chronology of the western steppes (Haak et al 2015; Allentoft et al 2015; Mathiesen et al. 2018; Damgaard et al 2018; Wang et al 2018; Narasimhan et al. 2019). Between 3100–2500 BCE the Yamnaya culture expanded out of its steppe homeland westward into central Europe, where people with ca. 70% Yamnaya ancestry created the Corded Ware horizon (Haak et al. 2015; Frînculeasa et al 2015; Nikitin 2018); and eastward to the Altai Mountains, where people almost identical to the Yamnaya population in genetic ancestry appeared as the Afanasievo culture (Allentoft et al. 2015; Narasimhan 2018). Today the origin and nature of the Yamnaya archaeological culture is a question with new relevance across northern Eurasia. Yet none of the key archaeological sites most important for understanding the evolution of Yamnaya customs and economy is published in a western language. These key sites would include Khvalynsk on the Volga (Eneolithic), Repin on the Don (EBA), and Mikhailovka (EBA) on the Dnieper rivers.^2^ This essay is about Khvalynsk, an Eneolithic cemetery on the Volga River dated ca. 4500–4300 BCE.

**Figure 1:**
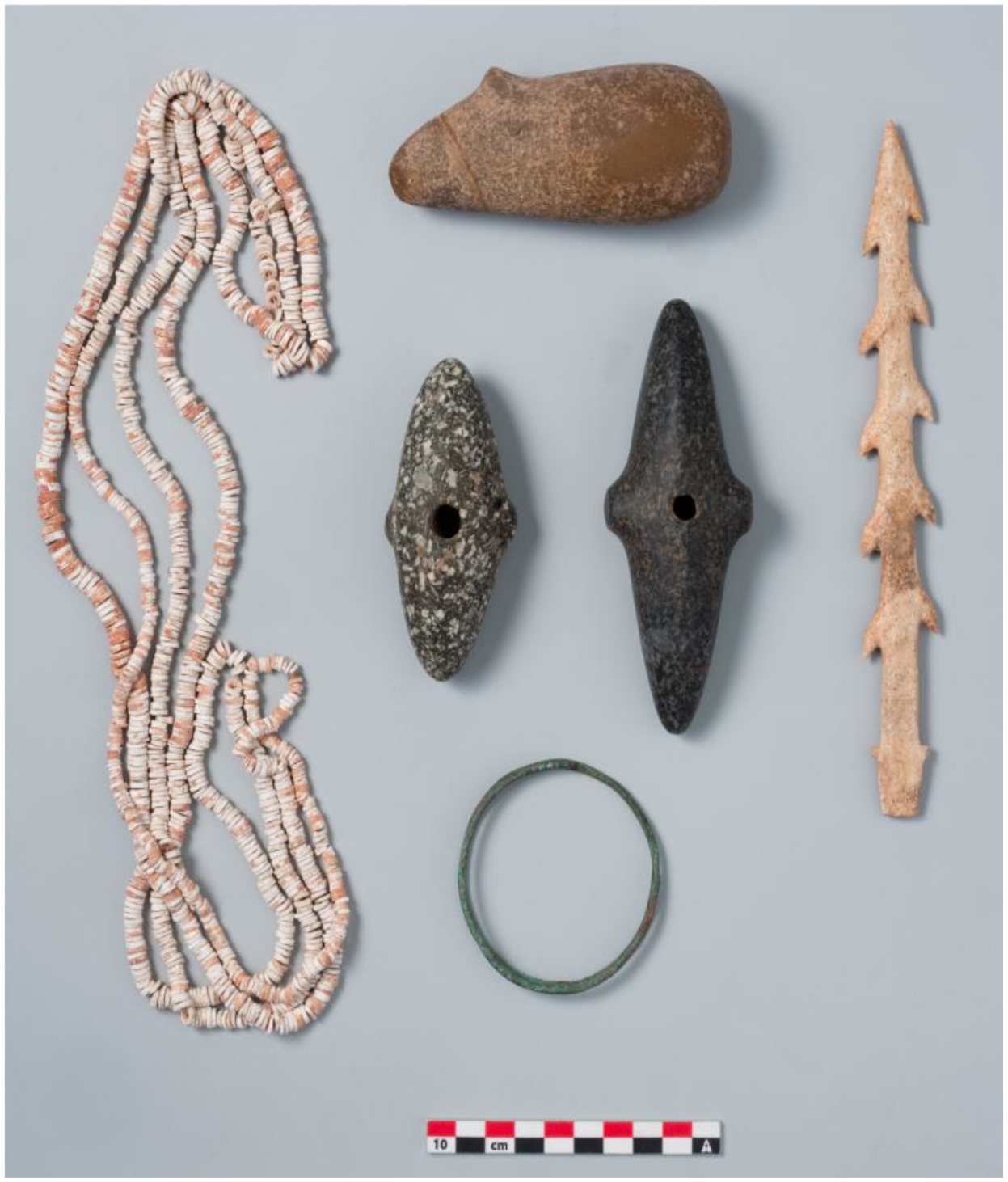
Artifacts from Khvalynsk I. Three polished stone mace heads, antler harpoon, copper bracelet, and Unio shell beads. Photo by the State Historical Museum, Moscow. Used with permission.

In this report we present new aDNA-based studies of the Khvalynsk population in combination with traditional archaeological and anthropological studies. The formal presentation of the ancient DNA data along with a comprehensive set of genetic analyses will be made in a separate publication; here, we summarize results where they are relevant to understanding the Khvalynsk cemetery population. In addition, we present specific analytical summaries of radiocarbon dates and stable isotopes, copper artifacts, animal sacrifices, and polished stone maces. We argue that Khvalynsk exhibits remarkable diversity in its population and equally remarkable segmentation between groups of individuals in its grave offerings (Figure 1). We discuss how the Khvalynsk cemetery was related to other sites and archaeological cultures during the steppe Eneolithic. We also discuss cranio-facial types, skeletal pathologies, and new data from aDNA on family relationships within the Khvalynsk cemetery, with broader comments on the evolution of “steppe ancestry” that later characterized the Yamnaya populations.

**Figure 2:**
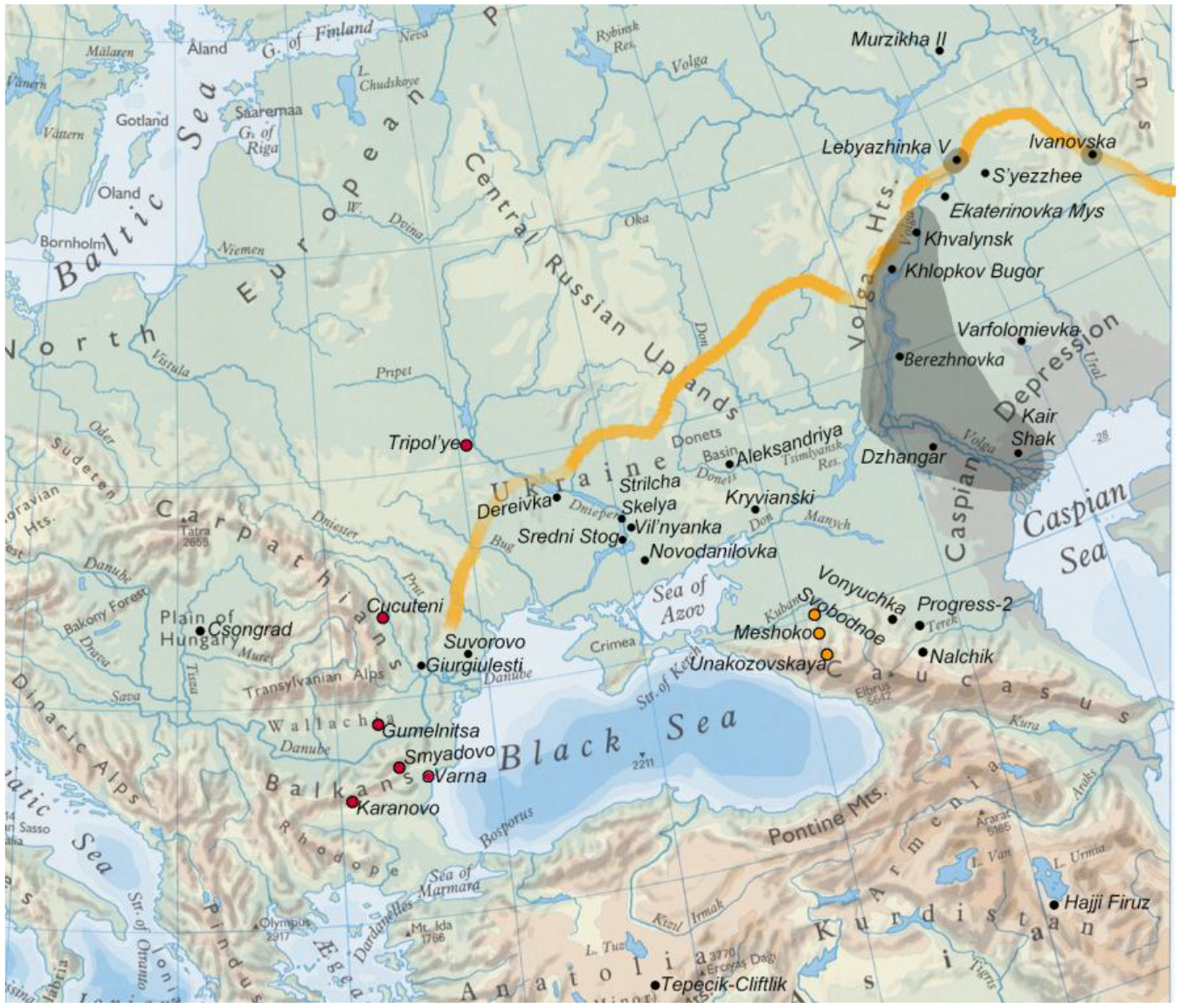
Important archaeological sites of the 5^th^ millennium BC in and near the Pontic-Caspian steppes. Orange line: ecological border of steppe. Black circles: steppe Eneolithic; red: Old Europe; orange: Meshoko Eneolithic. Stippled area: sites with Khvalynsk style ceramics.

Western archaeologists have asked if, by applying a cultural label such as ‘Yamnaya’ to a biological unit of analysis, as in ‘the typical Yamnaya pattern of genetic ancestry’, we imply that all Yamnaya-culture individuals had not only the same ancestry, but the same pottery, ornaments, and economy (Eisenmann et al. 2018; Furholt 2018, 2019; Hofmann 2019). However, to speak of a typical measurement or pottery type is never to imply that no outliers or variation existed; indeed, the concept of the average or typical observation implies the opposite. The Eneolithic populations of the Pontic-Caspian steppes (north of the Black and Caspian Seas) can be divided into regional groups defined by both material/cultural customs and genetic/morphological traits. Funeral customs (body pose, grave shape) varied regionally, as did pottery styles and economies. Ceramic types were more varied than cranio-facial or genetically defined groups. Figure 2 shows that Khvalynsk-style ceramics were the predominant ceramic type only in the middle and lower Volga steppes, while people with genetic ancestry like Khvalynsk can be found 1000 km to the south in the North Caucasus steppes. The Khvalynsk mating network extended far beyond the Khvalynsk pottery style.

Anthony refers to groups defined by similarities in their aDNA as mating networks (Anthony 2019: 2). A mating network is a population that shared a distinctive cluster of autosomal genetic traits such that individuals from that chronological period and region can be assigned to a space in a principal components analysis (PCA) plot that does not overlap significantly with the spaces occupied by other contemporary mating networks. Mating networks were maintained by the long-term, multi-generational exchange of daughters and/or sons as mates, creating significant gene flow between groups within the network. It cannot be assumed that mating networks were culturally relevant or even known to ancient populations, although borders between mating networks probably were recognized.

One interesting aspect of the Yamnaya mating network was its narrowness, more homogeneous (a more restricted space in a PCA) than the earlier Eneolithic populations. This reduction in genetic diversity between Eneolithic and EBA populations is interesting partly because EBA material culture (archaeology) exhibited contradictory trends at this transition—Yamnaya funeral rituals became more standardized and homogeneous than in the Eneolithic, like Yamnaya aDNA; but many regional Eneolithic artifact types, including regional ceramic types, continued into the EBA and appeared as regional variants that contrasted with the genetic and ritual homogeneity of Yamnaya ancestry and grave types. In the Pontic-Caspian steppes, mating networks and cultural traditions had a dynamic, changing relationship. Khvalynsk provides a data-rich window through which to examine the material and genetic variability of the pre-Yamnaya population at one of the largest and most important Eneolithic cemeteries.

## The importance of Khvalynsk

Khvalynsk is the largest excavated Eneolithic cemetery in the Don-Volga-Ural steppes (201 recorded graves). It has the largest copper assemblage of the late fifth millennium BC in the steppes (373 objects) and the largest assemblage of sacrificed domesticated animals (at least 106 sheep-goat, 29 cattle, and 16 horses). The human skeletons have been sampled extensively for ancient DNA, but genome-wide data from only three individuals has been published to date (Mathieson et al. 2018). Here we discuss relevant results from 32 analyzed individuals. The whole genomes of three additional Eneolithic individuals from graves in the North Caucasus steppes dated 4400–4100 BC at the Progress-2 and Vonyuchka-1^3^ cemeteries, broadly contemporary with Khvalynsk, were previously recognized as similar in ancestry and in PCA space to the published three from Khvalynsk (Wang et al. 2019: 3). This discovery expanded the range of the Khvalynsk mating network 1000 km to the south, from the Middle Volga steppes to the North Caucasus steppes. Unpublished samples from Volga Eneolithic cemeteries do not significantly alter the relationships or PCA space observed in the initial published samples, but rather form a cline between Khvalynsk and Progress-2. The Khvalynsk/Progress-2 ancestry cline represents a distant genetic ancestor, although not the exclusive or proximate ancestor, for the typical pattern of genetic ancestry exhibited in Yamnaya individuals. Yamnaya individuals cluster near Khvalynsk/Progress-2 in PCA space, but their distributions overlap only marginally (Wang et al. 2019; Narasimhan 2019). Yamnaya genomes had additional Anatolian Farmer ancestry (typical for agricultural populations in southeastern Europe and the North Caucasus) not present in Khvalynsk/Progress-2 (Wang et al. 2019: 6–7). Khvalynsk is important because of its size, its unique concentration of copper artifacts and domesticated animal sacrifices, and its genetic and ritual connections with the Yamnaya culture.

When Khvalynsk was discovered in 1977 it was recognized immediately, and with some excitement, as a good candidate for the elusive pre-Yamnaya archaeological phase in the Volga steppes (Vasiliev 1981). The shell-tempered, round-bottomed pottery of Khvalynsk, decorated with small comb stamps and shell-edge impressions, was like one of the earliest Yamnaya pottery types, found in Yamnaya kurgans in the southern Volga steppes (Bykovo I 12/7 type; see Mallory 1977). Also, the position of the body in most graves, on the back with tightly raised knees, was identical to a distinctive Yamnaya body pose often called ‘the Yamnaya position’ (Heyd 2012; Frînculeasa et al 2015). The abundant red ochre on the grave floor also was like Yamnaya. The differences between them (ornament and weapon types, absence of kurgans at Khvalynsk) were ascribed to the earlier chronological position of Khvalynsk (Agapov, Vasiliev, and Pestrikova 1990:83–85). It was anticipated that Khvalynsk would have radiocarbon dates in the early to middle fourth millennium BCE (Vasiliev 1981), not long before the oldest cluster of Yamnaya radiocarbon dates, 3300–3000 BCE. But radiocarbon dates calculated on human bone instead suggested that Khvalynsk dated ca 5200–4500 BC, almost 2000 years older than Yamnaya (Agapov et al. 1990:86–87). Archaeologists did not yet know that radiocarbon dates on Eneolithic human bones were skewed older by the absorption of old carbon in the bones of populations that regularly ate riverine fish, a phenomenon now known as a freshwater reservoir effect (FRE). The long chronological gap between Khvalynsk and Yamnaya was regarded uneasily as a ‘hiatus’ (Agapov et al. 1990:86–87; Rassamakin 1999:122). Here we show that Khvalynsk was in use about 4500–4300 BCE, about 1000 years before the Yamnaya culture appeared, contemporary with Skelya and early Sredni Stog^4^ in the Pontic steppes (Rassamakin 2002, 2013; Telegin 1986) and Varna I in the Danube valley.

A significant chronological gap still exists between Khvalynsk and Yamnaya. Subsequent discoveries of a few graves similar in ritual details to Khvalysnk and dated to the fourth millennium BCE have filled the gap to some extent, but the early fourth millennium BCE remains surprisingly poorly documented in the Volga-Ural steppes (Vasiliev and Ovchinnikova 2000; Morgunova 2014). This intermediate period is better documented in the North Caucasus steppes (Korenevskii 2016) and the Black Sea steppes west of the Don River, where the Sredni Stog culture introduced Khvalynsk-like grave rituals with Khvalynsk-like DNA traits 4500–3500 BCE (Kotova 2013; Rassamakin 2013). Sredni Stog is often seen as an ancestor of Yamnaya in Ukraine (Telegin et al. 2001; Anthony 2007: 240–249).

Other Eneolithic cemeteries were smaller than Khvalynsk, usually less than 30 graves. Khlopkov Bugor, 130 km south of Khvalynsk on the west Volga bank, had 24 excavated Eneolithic graves, including one person who was a 2^nd^-degree relative of a person buried at Khvalynsk (see below). A cemetery on the Volga 160 km north of Khvalynsk, at Ekaterinovka Mys, dated 150–200 years earlier, had more than 100 excavated graves (Korolev et al. 2018). Nalchik in the North Caucasus, approximately contemporary with Khvalynsk, had 121 graves (Gimbutas 1956: 53–54; Anthony 2007:187). A few other large Eneolithic cemeteries are known in the Volga-Caucasus steppes, but Khvalynsk was the largest.

The exceptional size of the Khvalynsk cemetery, as well the morphological (according to cranio-facial measurements) and genetic heterogeneity of those interred there, were linked to its integrative ritual position during an era of population movements and economic change in the Volga steppes. The Khvalynsk people showed ancestry from a southern population that can be derived, using both cranio-facial measurements and aDNA, from regions including the Caucasus and the lower Don steppes (Khokhlov 2017). These southern-derived people mingled in the Volga steppes, around Khvalynsk and south of Khvalynsk, with a population that can be derived, using both cranio-facial and aDNA data, from the northern forest zone (Khokhlov 2017). The admixed population that resulted from this north-south combination gathered at Khvalynsk to conduct funeral activities, feast on the meat of newly acquired domesticated animals, and celebrate alliance-making symbols (polished stone maces, see below). The earliest domesticated animals appeared in the middle Volga steppes around 4800–4600 BCE (using dates on animal bones), just 100–300 years before Khvalynsk (Morgunova 2015; Vybornov et al. 2019). Khvalynsk appears to have been a central place for the performance of these relatively new sacrificial rituals, a gathering place for genetically diverse populations participating in a new funeral cult focused on the ritual power and value of domesticated animals.

Copper ornaments were another introduced innovation used in a new way. Khvalynsk was part of a network of cultures that participated between 4500–4200 BC in the exchange of copper, exotic shells, domesticated animals, and emerging symbols of hierarchical leadership (polished stone maces) between the Volga steppes, the North Caucasus steppes, the Dnieper steppes, and the tell towns of the lower Danube valley and the Varna region in Bulgaria (Figure 2). The agricultural communities of the Karanovo VI/Gumelniţa/Tripol’ye B1 period were the copper-producing centers of the network, which Chernykh (1992) named the ‘Carpatho-Balkan Metallurgical Province’. Khvalynsk is the easternmost site included in this ‘province’ (Chernykh 2008). Khvalynsk is thus an essential site for understanding the introduction of domesticated animals and copper metallurgy to the steppes, and the genetic, morphological, and cultural origins of the population that would later become Yamnaya. Up to now, no English-language summary of the site exists beyond short descriptions contained in longer works (Anthony 2007: 182–186; Mallory and Adams 1997: 328).

## History and ecological setting of Khvalynsk I and II

**Figure 3.**
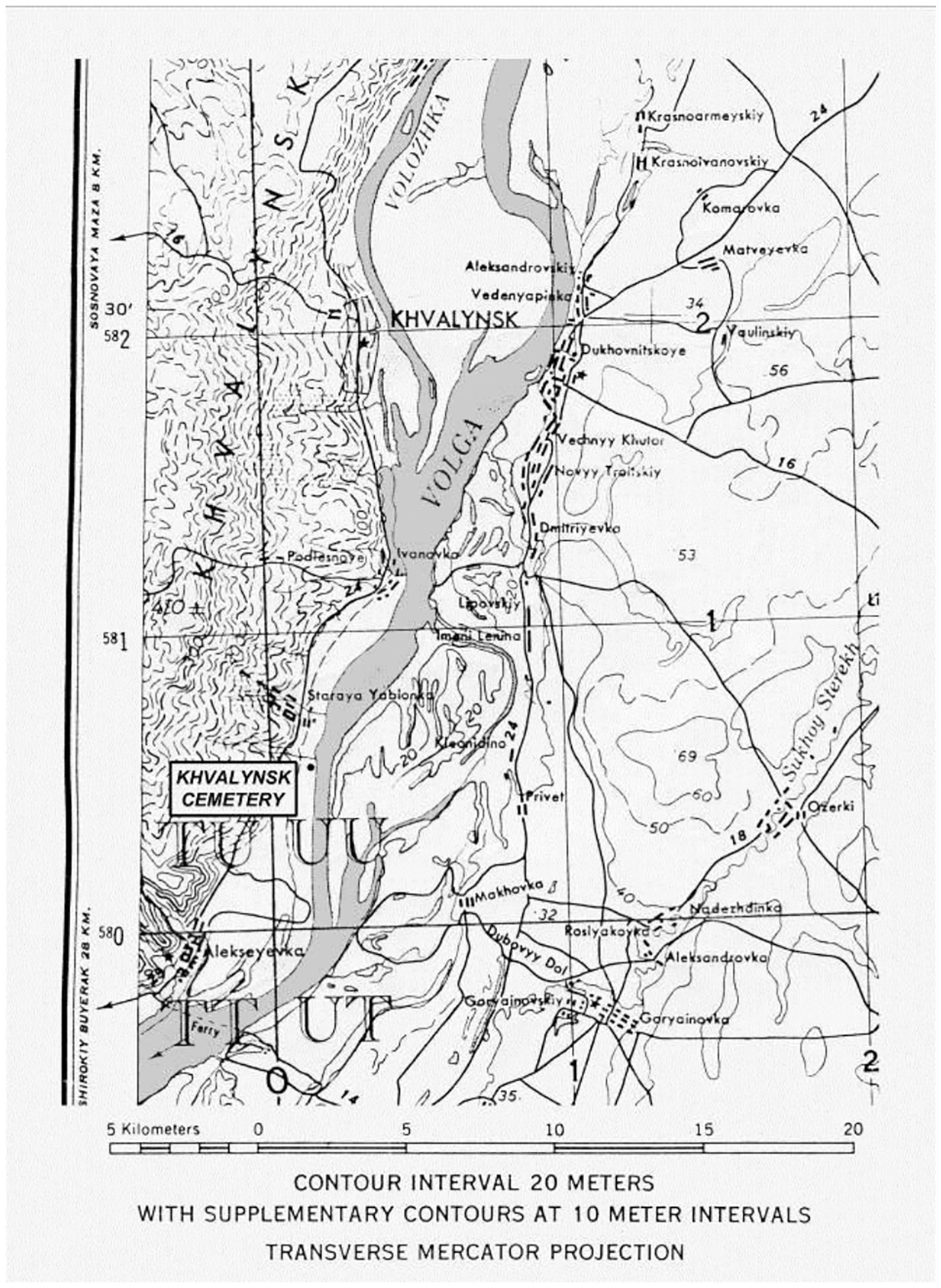
Topographic map of the Khvalynsk area ca. 1930 before dams, with cemetery marked. Army Map service, Corps of Engineers, 1957, Series N501, NN-3901, edition 3-AMS, 1:250,000; based on USSR 1:50,000 General Staff of the Red Army maps, 1929–1932.

Before 1971, when the hydroelectric dam was completed at Balakovo 40km downstream, the Khvalynsk cemetery was located on the west bank of the Volga River 16 km south of the town of Khvalynsk at 52°21’14.58”N, 48° 4’44.80”E (Figures 2, 3). The site is now under the Saratov Reservoir created by the dam. Here dry limestone hills overlooked the Volga on its west side, and a flat steppe plain rolled away to its east. The Eneolithic cemetery was at the foot of the western hills at a place where their white peaks stood 3–5 km away from the river. The Volga River, more than a kilometer wide, flowed through a wetland of large forested islands and marshes 5km wide at this location, now a 5km-wide reservoir (Figure 3).

Vegetation varied significantly between wooded ravines on the high west bank, a flat steppe on the east bank, and forests and marshes on the Volga bottomlands. At the Eneolithic settlement of Lebyazhinka VI north of Samara, with radiocarbon dates contemporary with Khvalynsk (Kulkova et al 2017), the most frequent fish bones were those of northern pike (*Esox lucius*), which in this region can weigh up to 25 kg; followed by catfish (*Silurus glanis*), up to 300 kg; and zander (*Sander lucioperca*), up to 20 kg (Kirillova et al. 2017). Large birds whose tubular bones were used for flutes or whistles in the Khvalynsk graves included white-tailed eagles (*Haliaeetus albicilla*), bustards (*Otis tarda*), cranes (*Grus grus*), and swans (*Cygnus sp*.) (Kirillova 2010:364). Broad-leaf forests on the 5-km-wide floodplain hosted moose (*Alces alces*), red deer (*Cervus elaphus*), roe deer (*Capreolus capreolus*), wild boar (*Sus scrofa*) and aurochs (*Bos primigenius*), as well as smaller game (Morgunova 2014: Tables 19, 20, 21). East of the gallery forest a dry steppe plain rolled unbroken to the Altai Mountains in Siberia. In the Volga-Ural region, the steppes contained wild horses (*Equus caballus*) in the northern steppes near Khvalynsk and saiga antelope (*Saiga tartarica*) and onagers (*Equus hemionus*) in the drier southern Volga steppes bordering the Caspian Sea (Morgunova 2014:Table 21; Vybornov et al. 2019).

**Figure 4:**
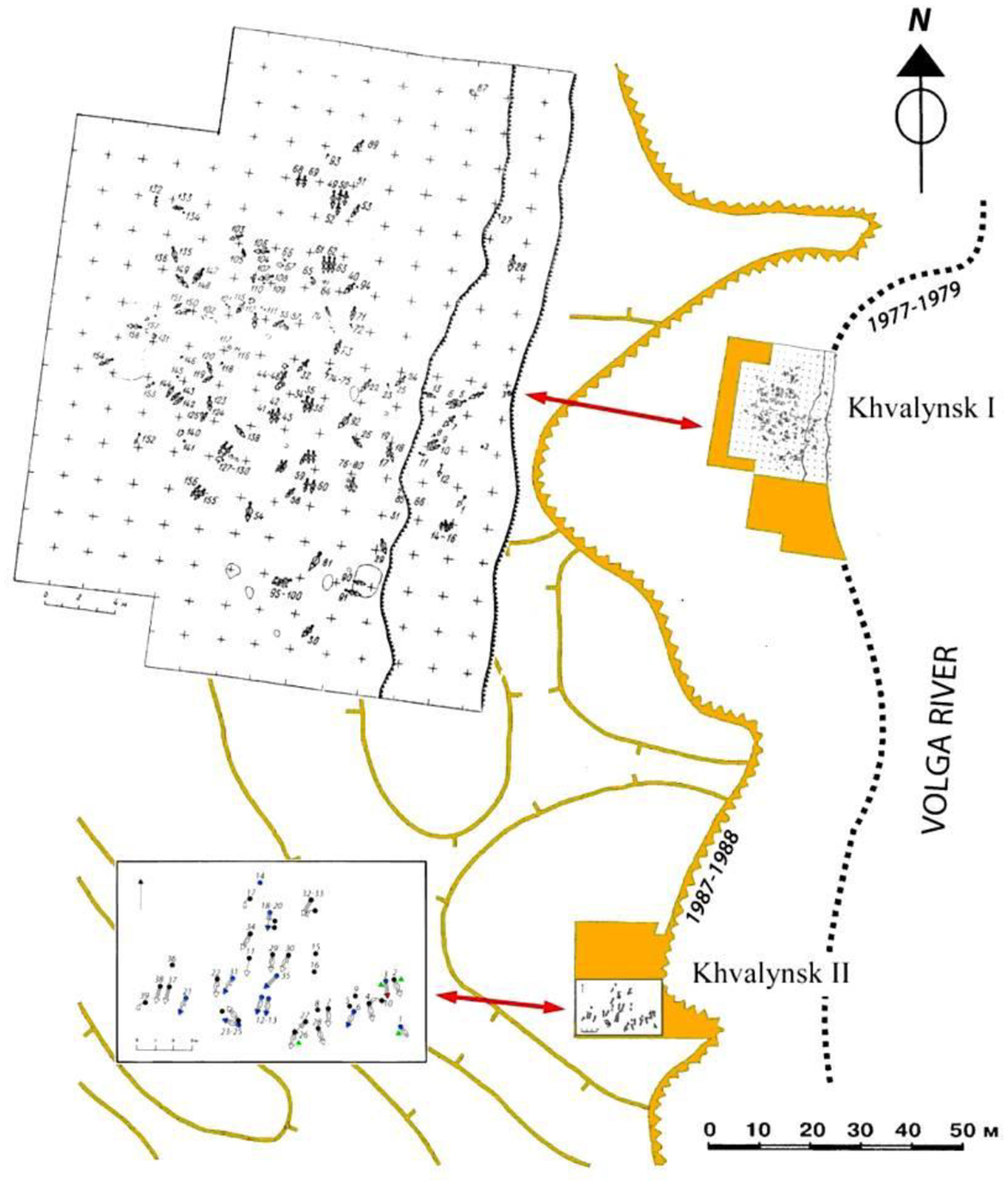
Plans of Khvalynsk I and II, with cemetery plans shown within the investigated areas (shaded). For II, the location of the cemetery-plan rectangle within the investigated area is approximate. (After Agapov Vasiliev & Pestrikova 1990: Figure 2; and S. Agapov 2010: un-numbered map on page 118.)

Erosion of the western bank after the reservoir was filled led to the discovery and salvage excavation in 1977–79 of the northern cemetery of 158 individuals, later designated Khvalynsk I (Figure 4). An unknown number of graves was lost before the archaeologists arrived. The director was I.B. Vasiliev, the energetic leader of the archaeology faculty at the Samara (then Kuibyshev) Pedagogical Institute, now the Samara State Social-Pedagogical University. The Volga continued to erode the cemetery during the excavation, swallowing 4m in the two years between 1977 and 1979, indicated in Figures 4 and 15 by the two parallel lines on the right side of Khvalynsk I. The graves were isolated in the center of the explored area, with no additional graves found to the north, south, or west, leading the archaeologists to believe that they had established the limits of the cemetery on all sides except the rapidly eroding east (Agapov, Vasiliev, and Pestrikova 1990:6–7). A report was published in the Russian language in 1990 (Agapov et al. 1990) describing each grave and surface sacrificial deposit.

Khvalynsk II was discovered 10 years after Khvalynsk I when continuing erosion exposed additional human skeletons 120m southwest of the first cemetery. Khvalynsk II was excavated in 1987–1988 by many of the same archaeologists from Samara (Kuibyshev) who had worked on Khvalynsk I, including A. Khokhlov and S. Agapov, co-authors of this report. I.B. Vasiliev again directed the first part of the excavation, and V.I. Pestrikova directed the second part. The Khvalynsk II excavation recovered 43 individuals (Khokhlov 2010:Table 1.1), again after an unknown number were lost to the Volga. A monograph describing both Khvalynsk I and II was published 22 years after the excavation ended (S. Agapov 2010). It contains many specialist studies (lithics, ceramics, metallurgy, fauna, shells, skeletal measurements, etc) in the Russian language. Pestrikova’s unpublished dissertation on Khvalynsk I was revised by D. Agapov for the opening essay of the 2010 monograph (Pestrikova and Agapov 2010). The 1990 and 2010 monographs are the most important sources of information for this report.

The funeral rituals (body pose, grave form, use of ochre, etc), ceramic types, lithics, ornaments, and radiocarbon dates from the two cemeteries are alike. Khvalynsk I and II were used during the same era by people from the same archaeological culture. But the demographic traits of I and II were sharply different. At Khvalynsk I males and females were buried in nearly equal numbers. Among 83 adults or adolescents to whom a sex could be assigned based on skeletal features, 45 (54%) were males and 38 (46%) were females (Khokhlov 2010: 410). Copper ornaments accompanied six adult males and five adult females at Khvalynsk I, again about equal, and were found with one infant and two adolescents.

The Khvalynsk II burial plot, in contrast, was heavily weighted toward adult males: more than three adult males for each adult female. Of 26 skeletons assignable to a sex, 20 (77%) were males and only six (23%) were females (Khokhlov 2010: 410). This was much like the proportion of males and females buried later in Yamnaya graves in the Volga steppes (80% males). This raises the question if Yamnaya gender practices evolved not from the preceding ‘culture’ but specifically from an Eneolithic sodality or other male-focused sub-group like the one buried in the Khvalynsk II cemetery. The absolute number of copper ornaments buried with 158 individuals at Khvalynsk I (35) was one tenth of the number found with 43 individuals at Khvalynsk II (338) (D. Agapov 2010). The abundant copper at Khvalynsk II accompanied about one third of both males and females: nine adult males and two adult females. Copper ornaments also were found with an infant and a child.

Khvalynsk I looks like a ‘family’ cemetery where all ages and sexes were buried, while Khvalynsk II was a more specialized cemetery for a copper-rich group of males (possibly warriors or traders), most of whom were related by family descent (see genetics section below), with a few unrelated males, females and immatures.

Unfortunately, another difference between I and II is that most of the skeletons from I were lost in a Volga River flood that destroyed the storage area where they were curated in the river-port city of Samara. One might say that the Volga reached for these graves twice. Contemporary studies, including ancient DNA studies, can be conducted only on male-dominated II and the small number of individuals from I that survived the flood (9 of 158—see Table 1 radiocarbon dates).

## Radiocarbon chronology and stable isotopes

Twenty radiocarbon dates were previously published from Khvalynsk: ten from Khvalynsk I and ten from Khvalynsk II (Table 1). Most were in Russian-language publications (Agapov et al. 1990: Table 5; Anthony 2007: Table 9.1; Shishlina et al. 2009; Chernykh and Orlovskaya 2010; Mathieson et al. 2018). Some dates were reported from the Soviet-era laboratory UPI, which operated briefly in Ekaterinburg in the 1980s, but never was included in the journal *Radiocarbon*’s global list of current and former radiocarbon laboratories. The 20 published dates are compiled here for the first time. Also, 28 new dates are presented here for the first time.

For Khvalynsk I, the ten dates previously published were from the Kiiv, UPI, and Groningen laboratories (Table 1). Three dates on shell beads are clearly subject to a variable freshwater reservoir effect; they are usually ignored in discussions of Khvalynsk chronology. Four new dates from the Pennsylvania State University Accelerator Mass Spectrometry laboratory (PSUAMS) are presented here, making 14 dates from Khvalynsk I. For Khvalynsk II, eight dates were published previously by the Oxford, Groningen, and Arizona laboratories, and two dates by PSUAMS (Mathieson et al. 2018). To these ten previously published dates we now add 24 new PSUAMS dates from Khvalynsk II, making 34 dates from Khvalynsk II (Table 1). Table 1 presents 48 dates for the Khvalynsk cemetery, 34 from Khvalynsk II and 14 from Khvalynsk I, including 28 new dates. In addition, Table 1 presents data on dietary stable isotopes (δ^13^C and δ^15^N) from 30 individuals, not all dated by radiocarbon.

Most of the dates are on human bones or teeth. This is a problem, because studies by Shishlina and van der Plicht have shown that radiocarbon dates from Eneolithic human bones can be more than 1000 years too old in this region, a result of freshwater reservoir effects (FRE) (Shishlina et al 2009, 2017). Therefore, most of the radiocarbon dates in Table 1 are skewed too old.

**Figure 5:**
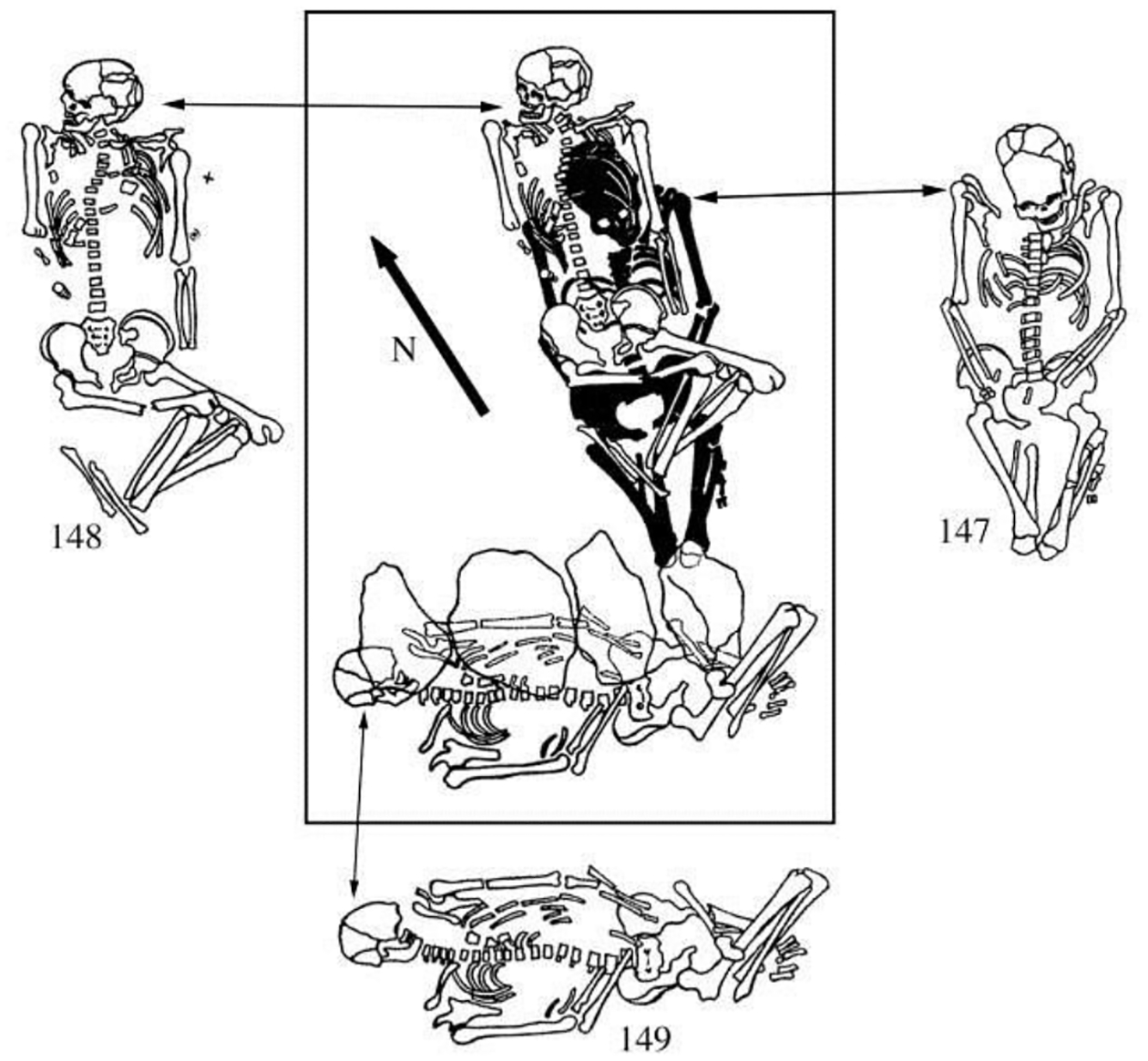
Skeletons 147–149 at Khvalynsk I. Individual 147, a female aged 40–50 with red ochre around her pelvis, had a bone ring made of sheep-goat bone that gave a radiocarbon date not affected by reservoir effects. Directly above 147 was 148, a male aged 30–40 with no red ochre, and at their feet was 149, a male aged 50–65 with no ochre, covered by flat stones. For a plan of the cemetery & location of 147–149 see Figure 15. After Agapov et al. 1990: Figure 22.

We have direct evidence of such skewing from two graves published by Shishlina et al. (2009), one at Khvalynsk I and the other at Khvalynsk II, with radiocarbon dates from domesticated cattle and sheep bones, not subject to reservoir effects (Shishlina et al. 2009: Table 8). Calibrated, the two samples produced statistically the same age: 4450–4350 BCE. A date of 4450–4355 BCE was obtained on a ring made of sheep bone (GrA-29178, 5565±40 BP) from grave 147 at Khvalynsk I (Figure 5). The human female buried with this bone ring was dated 4789–4618 BCE (PSUAMS 2886, 5845±25 BP), about 300 years older (Table 1). The second date, 4448–4362 BCE (GrA-34100, 5570± 40 BP), was obtained on a cow bone from grave 10 at Khvalynsk II. The human female in this grave was dated by Oxford to 4730–4530 BC (OxA-4311, 5790±85 BP), about 300 years older; but recently has been re-dated to 5210–5017 BC (PSUAMS 4149, 6150 ±25 BP), about 600 years older than the cow bone in the same grave (Table 1). It seems possible that a mistake was made in labeling one of these two human samples, but in any case, we can be confident that both came from Khvalynsk II.^5^

The offsets between faunal and human dates from the same grave indicate the presence of an FRE, in which consumption of aquatic resources (fish, shellfish, aquatic birds) leads to the incorporation of ‘old carbon’ into human tissues (Philippsen 2013). Therefore, the faunal dates of 4450–4355 calBCE from Khvalynsk I and 4448–4362 calBCE from Khvalynsk II provide the best estimate currently available for the true age of the two cemeteries. If we compare the midpoint of these dates, ca. 4400 calBCE, to the midpoints of the calibrated age ranges for the humans, the resulting offsets range between 43 yr (essentially no offset given the inbuilt uncertainty in radiocarbon dating) and 860 yr, with a mean FRE offset of 401 ± 288 yr (Table 1).

The details of the genetically-determined family trees at Khvalynsk are examined below (Figure 18). Here our narrow purpose is to use family relationships as a chronological check on the FRE connected with radiocarbon dates. In three of the five families at Khvalynsk II (Grey, Purple, and Orange), individuals who were nearly contemporary (1st to 3rd degree relatives) have ^14^C dates more than 100 years apart, and the older ^14^C dates are associated with lower δ^13^C values. In the extreme case (Orange), a father and son are dated minimally 247 years apart (between the 95% confidence intervals of the two dates), and the older date is linked to a lower δ^13^C value. The dates for related individuals confirm that the ^14^C dates at Khvalynsk II do not identify contemporary graves, so they are not reliable relative to each other. However, in some related pairs of individuals the older ^14^C date (indicating depleted ^14^C) is from the individual with lower δ^13^C values (indicating depleted δ^13^C).

**Figure 6.**
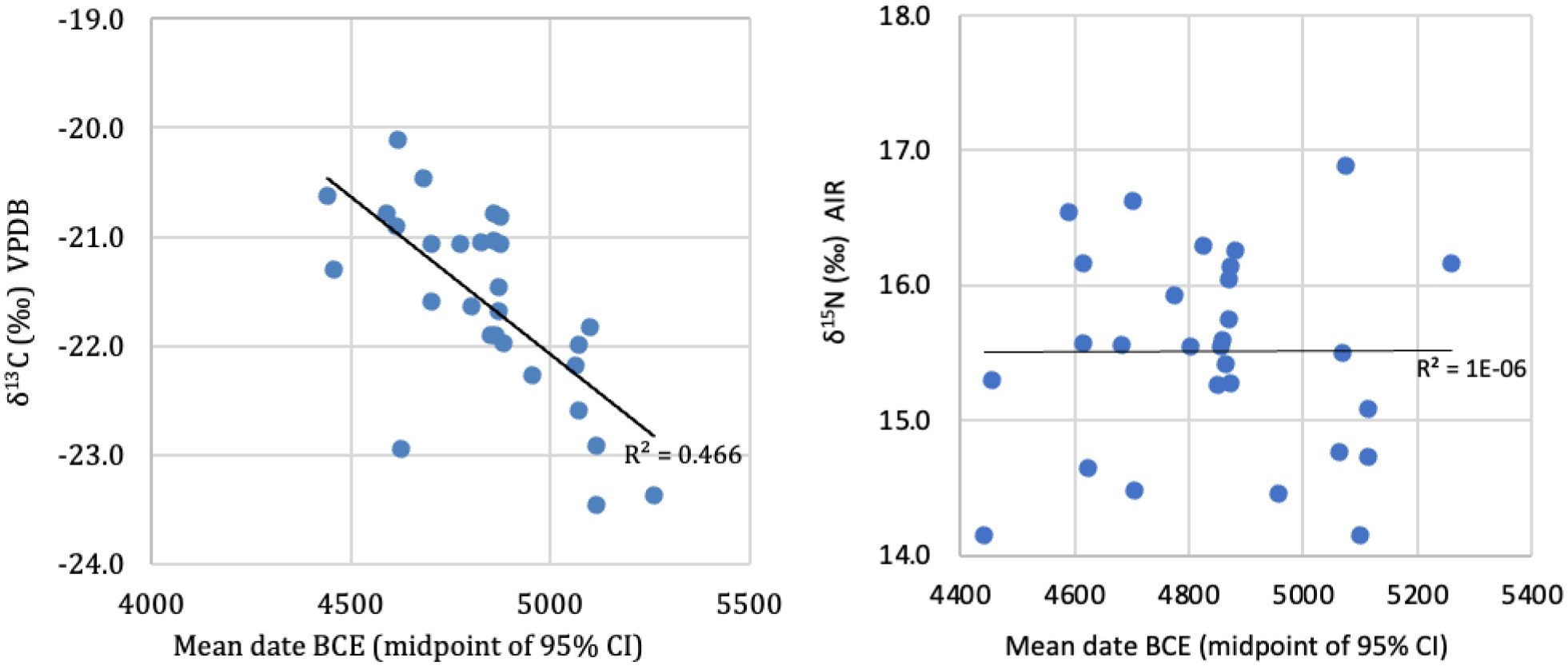
Human δ^13^C (a: left) and δ^15^N (b: right) bone/tooth collagen values plotted against the midpoint of date cal BCE (95% confidence interval, CI) for 29 individuals from Khvalynsk I and II. Note that removing the outlier in the lower left of Figure 6a significantly improves the regression (*r*^*2*^ = 0.663).

This is reflected in a moderate negative correlation between δ^13^C values and calibrated age, accounting for nearly half the variation in the latter (*r*^*2*^ = 0.474, *p* < 0.001, n = 29) (Figure 6a). There is a clear outlier (K-I, grave 17) with a predicted offset removed by nearly three standardized residuals from the assumed date of 4400 calBCE. Its removal improves the regression considerably (*r*^*2*^ = 0.663, *p* < 0.001, n = 28; the slope of the regression line remains similar). Extending the slope to the y-intercept suggests that a diet with no ^14^C offset would result in a δ^13^C value of ca. −20.3‰ (or ca. −20.0‰ if the outlier is excluded). In contrast, there is no relationship between δ^15^N values and calibrated age (*r*^*2*^ < 0.001, *p* = 0.995, n = 29) (Figure 6b). This is unexpected, since aquatic foods are typically significantly ^15^N-enriched compared to terrestrial flora and fauna (Anderson and Cabana 2007; Schoeninger et al. 1983), and therefore a positive relationship with radiocarbon offsets is often observed (e.g., Schulting et al. 2014). Since most of the analyses were made on the petrous bone, the core of which forms in infancy and does not remodel (Jørkov et al. 2009), it is possible that some samples retain a partial nursing signal (Schurr 1998), which could obscure the relationship between δ^13^C and δ^15^N values.

However, the relationship between radiocarbon offsets and both δ^13^C and δ^15^N values is complex (Cook et al. 2001; Higham et al. 2010; Wood et al. 2013; Fernandes et al. 2015; Svyatko et al. 2015; 2017; Svyatko, Schulting et al. 2017). Aquatic systems are often ^13^C-depleted, as seems to be the case on the Volga, but they may also be elevated relative to C_3_ terrestrial ecosystems (Dufour et al. 1999; Katzenberg and Weber 1999). And fish from adjacent watersheds, or even different parts of the same river, can exhibit variable ^14^C offsets, leading to different relationships with both stable isotopes (Fernandes et al. 2015, 2016; Svyatko et al. 2017). In the Upper Lena river system north of Lake Baikal, Siberia, a program of paired human–fauna dating from the same graves identified a comparable relationship in which ^14^C offsets (of up to 1000 yr) were better predicted by δ^13^C values than by δ^15^N (Schulting et al. 2015). This differed from Lake Baikal itself, where both isotopes were significant predictors, but δ^15^N accounted for the larger amount of the variability in ^14^C offsets.

The implication of the variability in the FRE at Khvalynsk is that individuals were acquiring aquatic resources from different catchments, subject to different ^14^C reservoir offsets. This is consistent with the cranio-facial metric and genetic data indicating that the cemetery served for communities of different origins to the south and to the north, though it places this within a context of the immediate lifetimes of individuals rather than their more distant ancestry. One possibility is that such access was held within families or clans, as was the case with the best fishing places on the salmon rivers of the Interior Plateau culture area of northwestern North America (Romanoff 1992). If so, we might expect to see a link between the genetic ancestry evidence and the FRE offsets.

**Figure 7.**
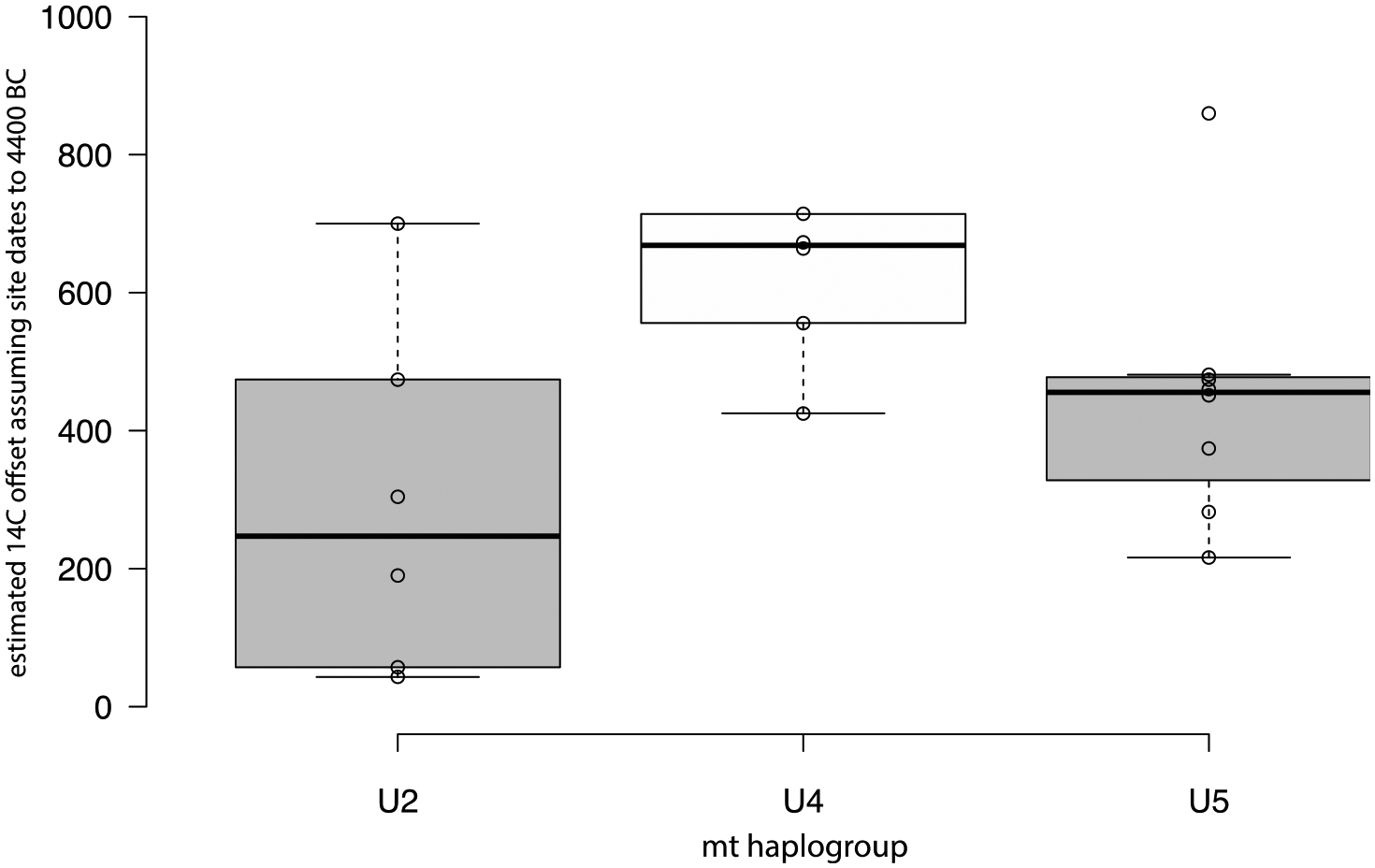
Boxplots comparing mean 14C offsets for mt-haplogroups U2, U4 and U5.

While this is not the case for Y-chromosome haplogroups, there is some indication of such a relationship between the estimated FRE offset and mitochondrial haplogroups. Limiting the comparison to haplogroups with more than five samples, the estimated mean FRE offsets relative to the faunal date of 4400 cal BC differ significantly for mt-haplogroups U2, U4 and U5 (ANOVA, *F* = 4.268, *p* = 0.031, n = 20). Bonferroni post-hoc tests show that the significant difference is between U2 and U4 (*p* = 0.029), with mean ^14^C offsets of 295 ± 256 yr and 624 ± 114 yr, respectively (Figure 7). Note that the same result would obtain if the means of the calibrated dates were used directly, since the same offset (i.e., from 4400 cal BC) is applied to all the individuals.

The Volga River appears to have been depleted in both δ^13^C and ^14^C in some of its catchments, creating a mild correlation between older ages and lower δ^13^C in the bones of people who regularly ate Volga fish from those parts of the river. The maternal mtDNA haplogroup U2 (represented in two lineages, U2e1b and U2e2a) differed significantly from U4 in its smaller average FRE offsets (with U5 being intermediate), perhaps suggesting that U2 females came from a riverine catchment with less depleted δ^13^C and ^14^C. Females with U2 maternal ancestry occur in both Khvalynsk I and II (Table 6). The richest grave at Khvalynsk II contained an older brother (Khvalynsk II:24) and a younger sister (II:25) who carried U2 mtDNA ancestry.

The mean faunal date of 4400 calBC probably is the most accurate estimate of the midpoint date for the Khvalynsk cemetery. A relatively short span of time is suggested by the fact that 70% of the individuals analyzed from Khvalynsk II were related to other individuals in ways that could fit within a 5-or-6 generation span, or about 140–170 years (Figure 18). Stable isotopes indicate a diet in which riverine fish played a large role, causing a strong FRE in radiocarbon dates on human bones and teeth. Variation in δ^13^C seems to identify Volga riverine catchments that were depleted in carbon. δ^13^C also correlated with mtDNA haplogroups, suggesting that females at Khvalynsk came from different riverine catchments, while the men’s Y-haplogroups did not display such patterning.

## Copper artifacts and trade

**Figure 8.**
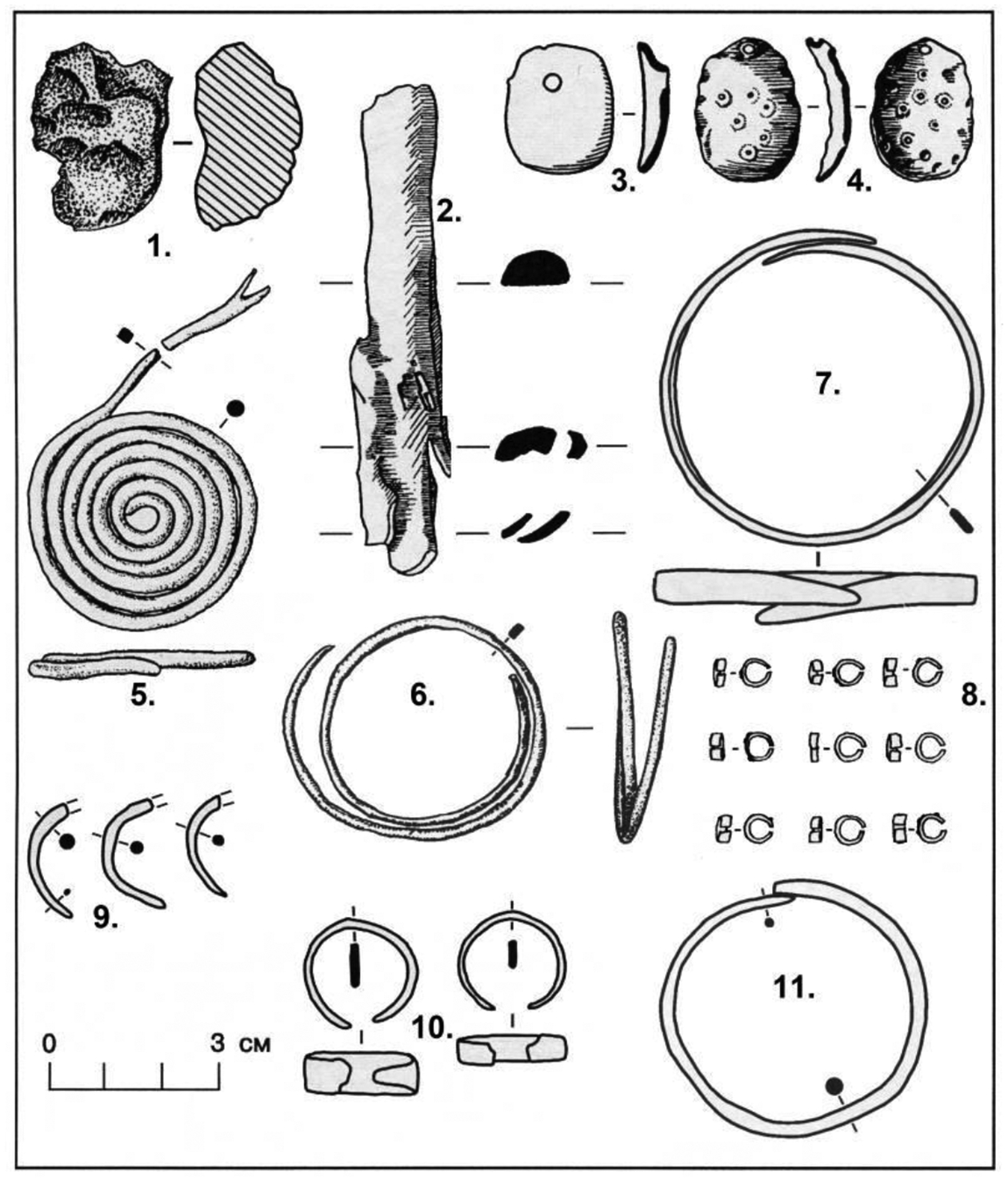
Copper objects from Khvalynsk II. *1*, grave 21; *2*, grave 24; *3*, grave 12; *4*, grave 35; *5*, grave 24; *6*, sacrificial deposit; *7*, grave 31; *8*, grave 12; *9*, grave 24; *10*, grave 24; *11*, grave 6. After D. Agapov 2010: Figures 8, 9, and 11.

The two Khvalynsk cemeteries together yielded 373 copper objects, the largest assemblage of copper items from any Eneolithic cemetery in the steppes. Almost all were ornaments (beads, rings, or bracelets) made of hammered sheet copper or wire, bent into tubes and rings. Four melted lumps of copper in two graves at Khvalynsk II (Figure 8:1 and Table 3) were possibly unshaped, primitive trade ingots or possibly were evidence of local production (but Khvalynsk pyrotechnology probably was not sufficient for production, see below). Two similar lumps, interpreted as ‘ingots’, were found at Khvalynsk I in the ‘cultural stratum’, but were not associated with a specific grave (D. Agapov 2010: 263). As noted above, the number of copper objects at Khvalynsk I (35) was one tenth of the number at Khvalynsk II (338). The count of 338 objects from Khvalynsk II includes 332 preserved objects (D. Agapov 2010: 258) and an additional six copper stains/traces that were recorded during the excavation but could not be catalogued (D. Agapov 2010: Table 1). Similarly, the count of 35 from Khvalynsk I includes one grave distinguished only by a copper stain. It is necessary to include the stains to identify the individuals who had copper objects. At Khvalynsk I, 9% of the individuals (15/158) had copper objects on or near their bodies, about one in ten; and at Khvalynsk II 30% of individuals (13/43), about one in three. Adding the two cemeteries together, 28 individuals (14% of 201) had at least one copper object.

Within the 14% minority that had access to copper ornaments, most had one to four pieces (Figure 8). A single bead of copper was an important find at Khvalynsk; presumably, it was just as important to the person who wore it. Most of the pieces were combined into sets such as beads strung together or connected rings made into a hanging ornament. One male aged 20–30 in Khvalynsk II: grave 12 was buried with 297 copper objects, most of them (293) simple copper beads strung on at least two necklaces also adorned with small sheet-copper oval pendants (Figure 8: 8 & 3). This single individual had 80% of the copper objects found in both cemeteries combined. If we exclude grave II:12 to see if it alone was responsible for the difference between Khvalynsk I and II, Khvalynsk II still would have 41 copper objects, more than Khvalynsk I (35) in one third the number of graves. A higher proportion of graves at Khvalynsk II (1/3 compared to 1/10) contained copper objects, so even without II:12 the two cemeteries differed significantly in their access to copper.

The copper-rich male in grave II:12 was the brother of the male in II:13, and the uncle of the male in grave II:22, who was the son of II:13 (*family relationships* below). The brothers in II:12&13 were the center of a cluster of seven related males that included II:4, II:7, II:22, II:27, and II:31 as second or third-degree relatives (the Yellow family in Figures 17 and 18). This patriline accounted for one of seven individuals at Khvalynsk II, the largest single family identified, and modeling described below suggests that the relationships within it should be distributed over four or five generations, so it was a persistent presence over more than 100 years. Its wealth in copper could have been related to its central position in the male-dominated group buried at Khvalynsk II. No female relatives—no mothers, daughters, sisters, or female cousins of the seven related men were buried with them. Khvalynsk II could have been a burial place for a multi-generational male sodality or society engaged in long-distance expeditions that brought Balkan copper to the Volga. The paternally central man in II:12 had much more copper than anyone else at Khvalynsk.

Family relationships also might suggest that the beginning of the Balkan copper trade occurred suddenly on the Volga, with copper changing from absent to abundant over the span of two generations, between grandparent and grandchild. The male in grave II:4 was a second-degree relative of a female in grave 7 at Khlopkov Bugor (KB7), a Khvalynsk-culture cemetery 130 km south near Saratov. No copper was found at Khlopkov Bugor, so it is generally thought to be older than Khvalynsk, although the artifact and ceramic types are quite similar. (We established above that their radiocarbon dates are variably affected by FRE and cannot be relied on to indicate their relative age.) If Khlopkov Bugor was older, then the Yellow-family female KB7 was a paternal grandmother or paternal aunt (given their different mtDNA haplogroups) of the male at Khvalynsk II:4. The chronological difference between them was no more than two generations, perhaps 50–60 years. If the absence of copper at Khlopkov Bugor is explained by its earlier position, then the copper trade began suddenly and abundantly when the Khvalynsk cemetery began to be used, about 4500 BCE.

**Figure 9:**
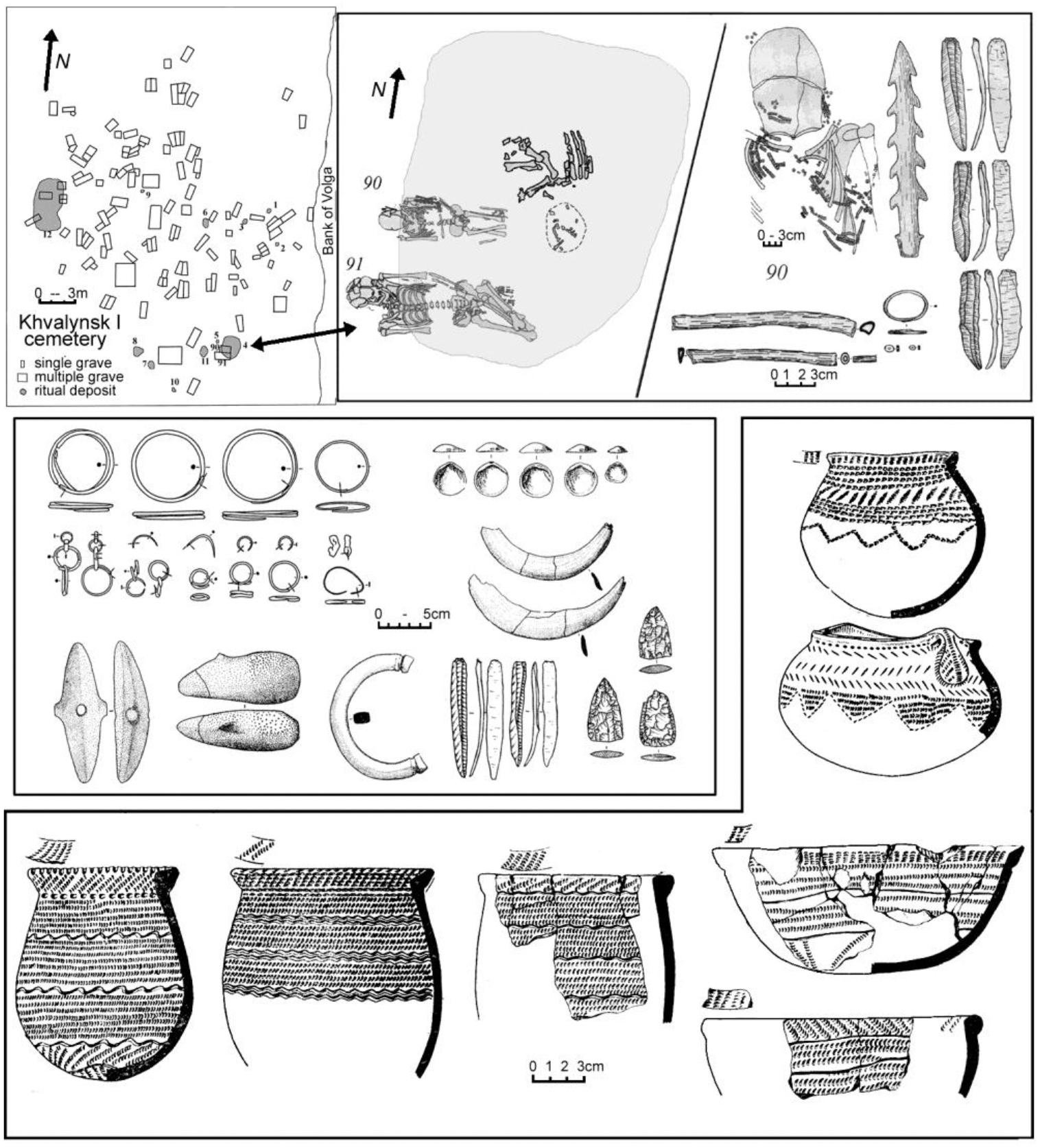
Khvalynsk I plan and objects. Top: cemetery plan and Sacrificial Deposit 4 containing bones of 2 cattle, 1 sheep-goat, & 1 horse above Graves 90 & 91 with bird-bone tube, harpoon (see Fig.1), flint blades, and copper ring. Middle: grave artifacts including the broken mace and whole mace from grave I:108, a polished stone bracelet probably from the North Caucasus, & fossil *Glycemeris* shell ornaments; Bottom: ceramic pots and bowls from Khvalynsk I. From Anthony 2007: Figure 9.7.

An artifact linked to the copper-using minority was the bird-bone tube, possibly used as a flute or whistle (Figures 9, top right; & 11). With one exception (II:4, the Yellow-family male related to KB7) bird-bone tubes appeared only in graves with copper ornaments, and only with adult males, or in one case, an adolescent buried with an adult male (I: 90 & 91, see Figure 9 top). They were not modified to create musical notes—they had no holes—so their function is uncertain. At Khvalynsk I, graves 19, 30, 57 (Figure 11), and 90 (Figure 9) had bird-bone tubes (Agapov et al. 1990: 60 n.), and Table 3 shows that all these graves contained copper ornaments with a male (or an adolescent). At Khvalynsk II, only grave 24 was described by the zoologist Bogatkina (2010: Table 1) as containing a bird-bone tube, and this was the richest grave at Khvalynsk, discussed below, belonging to a male equipped with many copper items. The Moscow zoologist Kirillova (2010: 363–366) found five more bird-bone tubes in collections that had moved to Moscow, from four graves at Khvalynsk II:4, 13 (two tubes), 18, and 35. All nine individuals in both cemeteries with bird-bone tubes were adult males (or an adolescent buried with an adult male), and all but one (II:4) were buried with copper artifacts (Table 3). It interesting that II:4 is modeled in the Yellow family relationships as the oldest Yellow family grave at Khvalynsk II, so perhaps the copper trade had not yet started when II:4 died. Kirillova specified that the bones were ulnas from large birds, which she tentatively identified as a swan, a white-tailed eagle, and three bones that were in the size class of swan-crane-bustard, among locally available large birds. Two of the three mace graves at Khvalynsk (see below) contained bird-bone tubes, which seem to have symbolized an office or status among the copper-using men (and one boy) at both Khvalynsk I and II. This restriction in the use of bird-bone tubes was one of many shared customs that connected I and II in the same ‘culture’.

Balkan ores probably were the source of the copper imported to Khvalynsk, although most of the imported metal was worked into rings and beads by local artisans. A Balkan source is surprising given the distance (2000 km) between Khvalynsk and the lower Danube valley. But ‘clean’ Balkan ores, specifically copper ores of groups B1-B2 and B3-B6 from Ai Bunar in Bulgaria, match the trace elements in Khvalynsk copper better than Caucasus ores do (Ryndina 2010: 242–243; D. Agapov 2010). Courcier argued that relatively ‘clean’ copper ores also were found in the Caucasus in some Chalcolithic artifacts, as at Menteshtepe (Courcier 2014: 596). But the Menteshtepe ‘clean’ copper had trace amounts of arsenic measured in the high tenths of one percent (range 0.6–0.9% arsenic). E.N. Chernykh analyzed 41 copper objects from Khvalynsk with methods capable of detecting arsenic, and only ten (12.2%) had any arsenic trace elements; more than 80% had no detectable arsenic. Of the ten exhibiting some arsenic, seven were in the range 0.0034–.1% (Chernykh 2010: Table 2), like the copper from Cucuteni-Tripolye sites, which ranged 0.007–0.1% (Chernykh 2010: Table 6). The trace elements in 70% of the tested Khvalynsk copper objects with arsenic fell into the range of the trace elements in Balkan copper rather than Caucasian copper. Three of the ten tested objects had arsenic outside the range of the tested Balkan copper objects, but not by very much: 0.2, 0.3, and 0.42. These three rings all were worn by adult females. Their slightly elevated arsenic might have resulted from a mixture with copper from Caucasian ores, so might indicate trade with the south.

**Figure 10.**
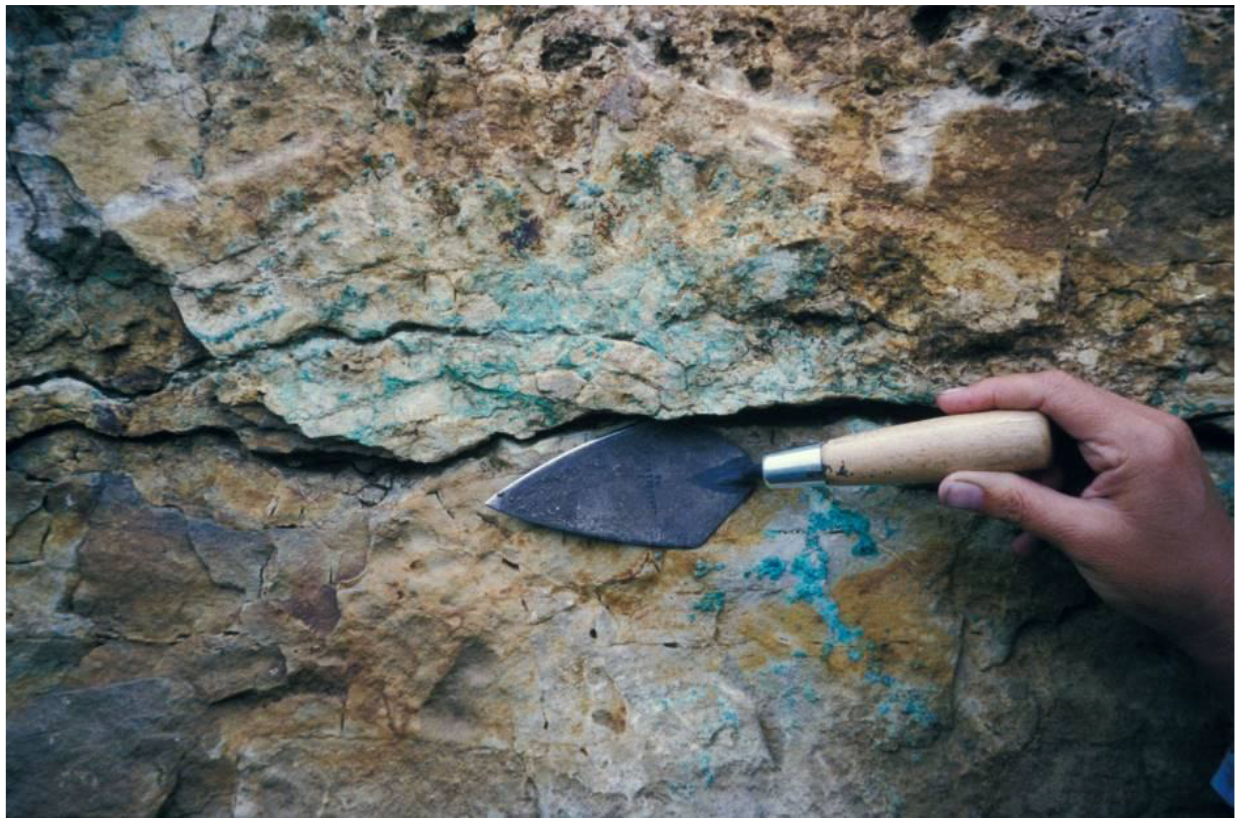
Multi-mineral sandstone copper oxide ore with malachite and azurite in an eroded ravine exposure 8km west of Mikhailovka Ovsianka (Mikhaylo-Ovsyanka in GoogleEarth), Samara oblast, Volga-Ural steppes. Mining began here in the Bronze Age. Photo by D. Anthony and D. Brown 2000.

**Figure 11:**
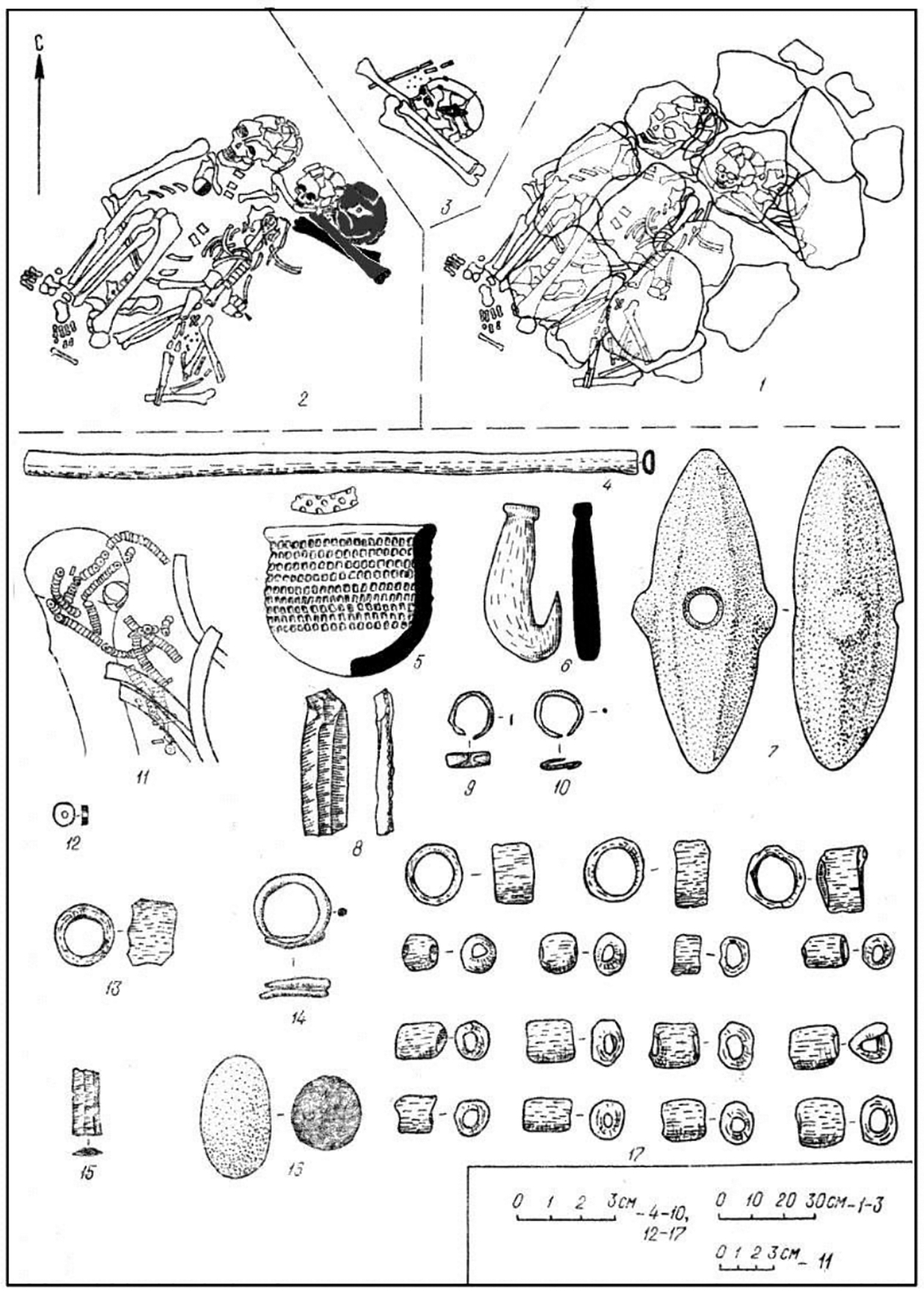
Mace chief grave, Khvalynsk I: 57. *1*, surface pavement of flat stones above skeletons 55–57. *2*, mace-chief 57 in black, beneath individuals 55 & 54. *3*, mace chief 57 with mace on skull. *Objects found with 57*: *4*, bird-bone tube. *5*, miniature ceramic cup. *6*, bone hook. *7*, polished stone cruciform mace. *8, 15*, flint blades. *9, 10, 14*, copper rings. *11,12*, *Unio* shell bead & string of *Unio* beads on humerus. *13, 17*, bone rings or large bone beads. *18*, abraded stone used as polisher. After Agapov et al. 1990: Figure 18.

‘Clean’ oxide copper ores are abundant locally in the Volga-Ural steppes (Figure 10), not far from Khvalynsk, but ore mining and smelting probably was not yet possible locally during the Eneolithic. To smelt copper from a multi-mineral sandstone ore usually requires charcoal heated to 1200–1300 °C, much higher than the maximum temperature (700–800 °C) attained in making Khvalynsk ceramics (Vasilieva 2010: 164). Khvalynsk pyrotechnology probably was not sufficient to smelt local copper oxide ores, which began to be mined in the Yamnaya period, by present evidence (Chernykh and Isto 2002). Eneolithic experimentation with metallurgy ultimately led to the beginning of extractive copper ore mining and productive metallurgy in the steppes during the fourth millennium BCE.

At least five copper ornaments examined by Ryndina were made at temperatures of 900–1000° C and must have been imported as finished objects; three of these were spiral rings like ornaments at Varna (Ryndina 2010: 239–240). But most of the other copper beads and rings were shaped at temperatures between 300–800° C, were rather crudely finished, and seem to have been bent and welded into shape locally (but using imported metal). Ryndina (2010) noted that the methods used for wire-making and welding on the Khvalynsk copper artifacts seem to have been copied after the methods used by Tripol’ye A and B1 metalsmiths, including the same welding method (adding a small strip of heated copper), but the Khvalynsk artisans used lower working temperatures, their work was cruder, and their welds often failed to join completely. One individual at Khvalynsk II: 21 was named ‘the smith’ by the excavators because his grave contained an unworked lump of copper (Figure 8:1), an antler hammer and a grooved stone hammer that might have been used to make sheet copper, and a beaver incisor that could have been used as an edge tool to cut sheet copper. He was not related to any of the known families at Khvalynsk II but had similar genetic ancestry.

Rassamakin (1999) proposed that the Dnieper Rapids region emerged in this era as a secondary center of ‘Skelya-culture’ metalworking between Varna and the North Caucasus steppes. Most of the Khvalynsk copper is consistent with this kind of secondary source, among local steppe artisans. This could also be the source of a copper bead found at Svobodnoe, made of Balkan copper (Courcier 2014). Svobodnoe was one of a series of agricultural settlements established in the Kuban River drainage after 4700 BCE by immigrant farmers who crossed the North Caucasus peaks from Georgia (Wang et al. 2019). They participated in the trading network that brought Balkan copper into the steppes. Svobodnoe also produced many polished greenstone axes with faceted butts, like the axe found at Khvalynsk in grave I:105, probably made in the North Caucasus. A polished serpentine bracelet at Khvalynsk found in grave I: 8 probably was made in the North Caucasus (Figure 9: middle panel); it was like bracelets at Nalchik. The Khvalynsk population was active in inter-regional exchange systems (Danube-Dnieper-Caucasus-Volga) that were stimulated by the heightened production of Balkan copper after 4500 BCE.

## Animal sacrifices: a new funeral cult

A complete zoological report on the Khvalynsk fauna has not been published, but partial descriptions are contained in four sources (Petrenko 1984: 48, 70; Agapov et al. 1990: 8–9, 60, 65, Figure 3, Tables 1&2; Kirillova 2010; Bogatkina 2010). These sources occasionally contradict each other. We arrived at the numbers presented in this text and in Tables 3 and 4 by following this rule: where one source contradicted another, Bogatkina (2010) was authoritative for the Khvalynsk II fauna, and Agapov et al. (1990: Tables 1&2) for the Khvalynsk I fauna. Bogatkina (2010) and Morgunova (2014: Table 18) attempted to re-count the Khvalynsk I fauna, but both gave numbers much smaller than Agapov et al. (1990: Tables 1&2). They apparently described only the Khvalynsk I bones that survived in the Samara laboratory in the early 1990s. The faunal data in the original 1990 report must be presumed to be accurate. That report had no separate chapter by the site zoologist, A.B. Petrenko, but she is credited on the first page where fauna is described (Agapov et al. 1990: 8), and the animal bones are identified to taxa and briefly described within the text by grave number or sacrificial deposit (bones found in ochre-stained deposits above the graves at both I and II). Summary tables of the fauna from the graves (Agapov et al. 1990: Table 1) and above-grave sacrificial deposits (Agapov et al. 1990: Table 2) provide only the number of individuals, not the number of bones, which was not reported for Khvalynsk I. Therefore, to compare I and II, we can use only the number of individuals, as in Table 2.

According to our interpretation of these sources, the animal bones recovered from Khvalynsk I and II represented the funeral sacrifices of at least 151 mammals. Three mammalian taxa were sacrificed: at least 106 domesticated sheep-goat (70%), 29 domesticated cattle (19%), and 16 horses (11%) whose domesticated status is debated. No obviously wild mammals were included in the funeral sacrifices, although wild species were represented in bone tools, ornaments, and flutes or whistles; and moose (*Alces alces*), red deer, horses, beavers, and fish were important in the diet at regional Eneolithic settlements (Vybornov et al. 2019; Morgunova 2014: Tables 19 & 20). At the Eneolithic Ivanovska settlement on the upper Samara River, dated 4360–4220 BCE (68%) (Ki-15086 5440±80 BP), with pottery of the ‘Samara’ type, distinct from Khvalynsk pottery, horses contributed 40.2% of the 6068 animal bones, domesticated cattle 11.4%, domesticated sheep-goat 7%, moose 17%, and beaver 22.5% (Morgunova 2014:Table 19), not counting fish or birds. Sheep-goat were ten times more frequent in the funeral deposits at Khvalynsk than at the Ivanovska settlement. However, in seasonal (winter?) camps containing Khvalynsk pottery on the lower Volga, as at Kair-Shak VI, dated 4400 BCE, sheep-goat were 60–70% of bones, and wild saiga antelope and onagers were 15% (Vybornov et al. 2019: Table 2). The sacrifices at Khvalynsk did not include the wild game animals that were prominent in the diet at both settlements. Instead, domesticated mammals were exclusively used to communicate with the spirit world.

What segments of domesticated mammals carried the prayers of the mourners? At Khvalynsk, horses were represented by one or two bones of the lower leg, usually a single phalange, in contrast to cattle and sheep-goats, which were represented by head *and* lower leg bones. Most of the described elements for cattle and sheep-goat were distal leg bones (principally metapodials and phalanges) and skulls, mandibles, or teeth (Agapov et al. 1990; Bogatkina 2010). Head and leg bones might be the result of ‘head-and-hoof’ deposits, in which the skin or hide of the animal with head and hooves attached is left at a ritual site as the symbol of the gods’ portion, while the meat is consumed by the human participants. Head and hoof deposits occurred throughout Eurasian steppe prehistory and into the modern era (Piggott 1962; Taylor et al. 2020). In the Eneolithic they are indicated at Khvalynsk and at another late 5^th^ millennium BCE cemetery on the Samara River, a tributary of the Volga, at a site known as S’yezzh’e, containing ‘Samara’ style pottery, like Ivanovska. At S’yezzh’e parts of two horse heads and distal legs were found in an ochre-stained sacrificial deposit above nine Eneolithic graves, arranged in head-and-hoof offerings like the cattle and sheep-goats at Khvalynsk (Vasiliev and Matveeva 1979).

Where were the sacrificed animals deposited? About two thirds of the animal sacrifices at Khvalynsk were found in graves, associated with individual humans. These animal bones were connected to individual human deaths. One third of the sacrifices were in red-ochre-stained sacrificial deposits above the graves, possibly not connected with individual deaths but rather conducted for the public (Table 2, Figures 9 & 15). The sacrifices at S’yezzh’e were like these, in a red-ochre-stained deposit above the graves. Eleven of the 13 sacrificial deposits at Khvalynsk I (85%) contained the bones of domesticated sheep-goats, domesticated cattle, and/or horses, the same three taxa found in the graves. The two sacrificial deposits that did not contain these taxa (SD 9 & 13) contained a greenstone adze in a red ochre deposit in SD 13, and a bird (not identified) skeleton decorated with two shell beads and one copper bead lying on a red-ochre-stained bark plate in SD 9. Domesticated animals and horses were the *exclusive* mammalian sacrificial offerings in the sacrificial deposits as well as in the graves at both I and II.

The inclusion of horses in graves with humans and domesticated animals, and the equally interesting exclusion of obviously wild animals such as moose, suggests that at Khvalynsk the symbolic status of horses had started to move toward the domesticated pole on the wild-domesticated continuum by 4500 BC. Horses were treated like domesticated animals in three ways: they were buried with humans and domesticated animals in graves that excluded obviously wild animals; at S’yezzhe they were arranged in head-and-hoof deposits like the cattle and sheep-goats at Khvalynsk; and horse images were new symbolic artifacts. Decorative bone plaques shaped like horses were found at S’yezzhe and zoomorphic mace-heads that might represent horse heads were found at Khlopkov Bugor, 130 km south of Khvalynsk; and at Lebyazhinka IV, an Eneolithic settlement near Samara (Kriukova 2003). The evidence for a significant change in the human treatment of horses during the fifth millennium BC is symbolic rather than zoological, but it should not be ignored. In addition, recent studies of ancient horse DNA (Librado et al. 2021) indicate that the horses in the Don-Volga steppes in this era were the genetic ancestors of the modern domesticated horses that first appeared in fully modern form about 2200–2100 BCE in the Don-Volga region. The symbolic changes in the human treatment of horses seen at Khvalynsk, S’yezzhe, and other Volga sites signal the earliest phase in an experimental selection process between humans and horses in this region that produced a gradually improving partnership over the next two millennia, culminating in horses genetically and behaviorally suited for warfare, like modern horses. Perhaps the Khvalynsk horses could be trained to ride in quiet settings such as herding.

The proportion of individuals buried with domesticated mammal sacrifices was 14% at Khvalynsk I (23 of 158 individuals) and 14% at Khvalynsk II (6 of 43 individuals). Counting only adults preserved well enough to be assigned a sex, the percent receiving sacrifices was higher: at Khvalynsk I, seven females had an animal sacrifice, or 18% of adult females; and 12 males, 27% of adult males; four immature individuals also received animal sacrifices. At Khvalynsk II, animal bones occurred with two adult men (II:38 and the mace chief II:24, together 10% of adult males), three females (50% of females), and two immatures (Table 3, Figure 17).

The largest single sacrifice associated with a specific grave was the complex grave in Khvalynsk I:142–144, where two adult men aged 45–60 and 30–40 and an adult woman aged 40–50 were buried together on their backs with tightly raised knees (see Figure 15 for cemetery plan). With them were a first phalange of a horse and the skulls of eight cattle (Agapov et al 1990: Table 1). We can estimate edible meat weight as about 40% of adult body weight—for example, a 500 kg steer yields about 200 kg of ‘retail’ meat. Neolithic domesticated cattle in eastern Europe weighed between 350–500 kg (Kyselý 2016:44); let us use 400kg. If the cattle at Khvalynsk weighed 400 kg, eight cattle would produce 1280 kg of meat, and the horse another 120 kg (assuming a pony-sized body weight of 300 kg), equaling a total meat weight of 1400 kg. for the mammals in I:142–144. While no precise estimate is possible, this quantity of meat implies that the guests numbered in the hundreds. At Khvalynsk II, a similar large sacrifice was found in a sacrificial deposit above the graves in Quadrat I/8 (Bogatkina 2010: 400). This deposit contained heads and hoofs of at least two cattle and two sheep-goats, and the lower limbs of two horses. These animals again would have yielded around 1400 kg of meat, like the large sacrifice at Khvalynsk I, and again imply hundreds of guests. Large-scale feasts are implied by the large mortuary animal sacrifices at Khvalynsk.

Five hallmarks of competitive feasts conducted to create and maintain socio-political power, according to a recent analysis of feasting by Kassabaum (2019: 614–615), are large quantities of special foods shared between large groups at special places in the presence of special markers of elite status (maces and copper, here). In kin-based societies with competitive sections, feasts are an important arena for competition between lineages and clans (Hayden 2012: 126), while at the same time they channel that competition into non-violent rituals that often play an integrative, peace-making role (Dietler and Hayden 2001).

The feasts associated with funerals were sponsored or channeled through 14% of the population, and the animals sacrificed were not representative of the complex diet of fish, wild game (moose and deer), horses, and domesticated mammals that characterized everyday food consumption in Eneolithic settlement faunas in the middle Volga region (Morgunova 2014: Table 20; Schulting and Richards 2016; Vybornov 2019). Domesticated mammals, segmented and represented in funeral rituals by their parts, were used at Khvalynsk as a ritual currency to mark and symbolize social segments among the funeral guests and their families. Males, females, children, and even infants were among the designated minority to receive sacrifices. The status connected with mortuary mammal sacrifice seems to have resided in multi-generational families or in the role played by the sacrifice receiver in the funeral ritual rather than in the lifetime accomplishments of the deceased.

Domesticated animals, first adopted in the Volga-Ural steppes about 4800–4600 BCE, had triumphed by 4500 BC as the principal means of communication with the gods and ancestors, who apparently desired only sheep and goats, cattle, and an occasional horse. The horse was the only acceptable mammal that was indigenous. This new system of belief about the desires of the spirit world necessarily post-dated the arrival of domesticated animals, so it was a recently established ritual in 4500 BC. Yet this was the *exclusive* sacrificial ritual in the funerals at Khvalynsk. Khvalynsk was a central cemetery (because of its size) for a new funeral cult in which domesticated animals were the preferred channel of communication with the spirit world. If the Volga steppes were part of the Proto-Indo-European homeland, as many have argued (Anthony and Ringe 2015; Reich 2018; Narasimhan et al. 2019) then from the point of view of Indo-European religion, this was the moment when the world, made from the pieces of a cosmic cow (Mallory and Adams 2006: 435–436), began.

## Four depositional groups: social segments at Khvalynsk

At least four depositional groups can be identified archaeologically at Khvalynsk. Three were defined by the presence of copper, animal sacrifices, and polished stone maces in graves; and the fourth, the majority, by their absence. The ca. 70% of graves that contained neither copper nor animal sacrifices nor maces did contain some notable bone, stone, and antler artifacts and many beads made of exotic imported shells.

About 14% of the population was buried wearing copper ornaments, and a different 14% with sacrificed domesticated animals. It is remarkable that these two minority groups were so similar in size *and* that they did not overlap more than expected, if the deposition of grave goods had occurred at random. An excessive overlap might be expected if people of higher social status had an elevated probability of receiving both types of grave goods, but with a few important exceptions noted below, the two groups were separate.

At Khvalynsk I, among 158 excavated individuals, 34 (17%) were buried with copper and/or mammal bones. In 32 of these 34 cases (94%), copper and sacrificed animal parts occurred separately, with different individuals (for supporting data see Table 3 and Figure 15). Copper ornaments occurred *without* animal bones with 13 individuals (8% of 158), and animal bones *without* copper with 20 different individuals (13%). Both exceptions at Khvalynsk I, two adult males with both copper and animal sacrifices (I:57 and I:108–110), also had polished stone maces, and in fact were the only individuals at Khvalynsk I with stone maces, suggesting that apart from the stone mace holders (see below), there was a disassociation between copper users and sacrifice receivers.

Khvalynsk II exhibited a similar separation between an animal-receiving minority and a copper-receiving minority, but with much more copper in the graves. Here out of 43 excavated individuals, 19 (44%, more than 2x the percentage at Khvalynsk I) had copper artifacts and/or animal sacrifices. Copper ornaments occurred *without* animal bones in the graves of 13 individuals, the same absolute number found at Khvalynsk I. These included nine males, two females, and two immatures. Animal sacrifices occurred *without* copper with three different individuals, one male, one female and one immature. In 16 of the 19 graves that had copper and/or animal sacrifices—84% of cases—they again occurred separately. The remaining three cases at Khvalynsk II where copper and animal sacrifices occurred in the *same* grave were divided between a richly equipped adult female (II:6), the isolated skull of an infant (II:14) buried with a string of copper beads and two horse phalanges, the only horse bones at Khvalynsk II; and an adult male with a stone mace (II: 24), the richest grave at Khvalynsk, discussed below.

At both Khvalynsk I and II, people buried with animal sacrifices did not in general have copper ornaments, and people who wore copper ornaments into the grave did not in general receive animal sacrifices. (Bird bone tubes are counted as an artifact, not a sacrifice.) The segregation between these groups is surprising. Under a model in which higher social status confers an elevated chance of receiving both offerings, they should have overlapped more. Instead, their segregation suggests that they represented distinct statuses. The copper ornaments were worked and welded locally, but the metal probably was obtained from Balkan cultures where smelting was practiced. Copper represented contacts with distant others. It symbolized foreign adventures and long-distance travels (which also can be seen in some cultures as journeys to ancestral worlds, see Helms 1992). The mammals for sacrifices were, in contrast, herded and produced nearer to Khvalynsk, so came from and symbolized a different set of locations and behaviors. Animal sacrifices were sacral (connected with funerals and spirits of dead ancestors) and local. A sacrifice was shared during integrative feasts attended by hundreds. Copper ornaments, in contrast, were deployed on the bodies of specific individuals, a minority, presumably with pride and its companion envy. A feast animal was partible and belonged at least temporarily to everyone, while a shining metal ornament decorated the individual who wore it.

It is tempting to interpret the sacrifice-receivers as members of a local sacral group such as shamans or priests (and their families) who were buried primarily at Khvalynsk I; and the metal-users (with their bird-bone whistles) as members of a male-biased, far-ranging group, such as traders or warriors, defined by their visits to different cultural worlds and/or access to metals obtained abroad, buried primarily at Khvalynsk II. Horseback riding perhaps already facilitated long-distance travel. Sacrifice-receivers were buried at Khvalynsk II, but it is interesting that none of them except the mace chief was related genetically to any other person at Khvalynsk II, while many of the copper-receiving males were related to other males in that cemetery. As a cemetery, Khvalynsk II was organized around related copper-receiving males, while the sacrifice-receivers were perhaps wives or sacrifices themselves (the infant in II:14). Polished stone maces identified a special class of leaders who united these two groups, and occurred in both cemeteries, implying that leadership was not limited to one cemetery or to one of the minority groups.

## The mace-holders: Eneolithic chiefs

Only three individuals among the 201 excavated at both cemeteries had a polished stone mace (I:57; I:108; and II:24). One was the adult male in Khvalynsk I:57, the only adult male with copper objects *and* an animal sacrifice among 158 individuals at Khvalynsk I. This rare combination was recognized by a polished stone mace. The second mace holder was another adult male buried in grave 24 at Khvalynsk II with the most diverse and numerous assortment of artifacts at Khvalynsk, including an animal sacrifice (a sheep and a goat) and a large number of copper objects (Figure 8: 2,5,9,10). Like I:57, he was the only adult male at Khvalynsk II who had *both* copper objects and an animal sacrifice; and he was distinguished by the only polished stone mace. The third mace holder deviated from this pattern. Grave 108 at Khvalynsk I contained an adult male buried with two polished stone maces, one broken and one whole. Of the three mace graves, his was the only one without copper ornaments, animal sacrifices, or a bird-bone tube. He was buried in a cluster of four individuals (I:108–110), the other three all immature. One had an animal sacrifice and another had a copper item, so together they had the animal sacrifice+copper+ adult male combination that distinguished the other two mace graves. The difference is that in I:108 the mace holder did not unite these categories within himself by combining them. Let us look more closely at these three graves.

The mace in I:57 (Figures 1, lower right; and 11:7), was deposited on the skull of a male aged 40–50, whose red-ochre-painted skull and long bones were buried in a complex grave. His was a secondary burial, including only his skull and some long bones. With these were strings of bone rings, bone beads, and *Unio* shell beads; three copper rings (copper-user); two unifacial flint blades; a miniature ceramic cup (several other miniature cups were included in graves at Khvalynsk); a large bone fish-hook; an abraded stone polisher; and a bird-bone tube. On top of his red-ochre-painted skull was the polished stone mace-head. The burned skulls and lower limb bones of one *Bos* and one *Ovis* (Agapov et al. 1990: 29, Table 1) were found near his skull (sacrifice-receiver). These animal bones were burned outside the grave—the grave itself showed no signs of fire—then were placed in the grave. The meat from these animals, around 170 kg, was sufficient for more than 100 mourners.

The remains of four other people were placed above individual 57, under a stone pavement: a male aged 40–50 intensely colored with red ochre, and an adolescent aged 13–14 without red ochre, on their backs with tightly raised knees (I:55 and I:56); and mingled with 55 and 56, the isolated bones of a woman aged 40–50 (not assigned a number) and a child aged 6–10 (I:36), curated and re-buried, like the mace-chief. Because these bones do not survive, we do not know if the five individuals in grave 55–57 were related genetically. The bones of the mace chief, a woman about his age, and a child seem to have been curated until the deaths of 55 and 56, when these two were buried ‘in the flesh’ with the curated bones of the other three. The remains of the mace chief were interred with his mace, ornaments, and the head-and-hoof remains of a funeral feast; then 55 and 56 were posed above him, 55 being placed on soil stained with red ochre; then the curated bones of the woman and child were scattered over the bodies of 55 and 56; and finally a pavement of flat stones was placed above the grave.

**Figure 12.**
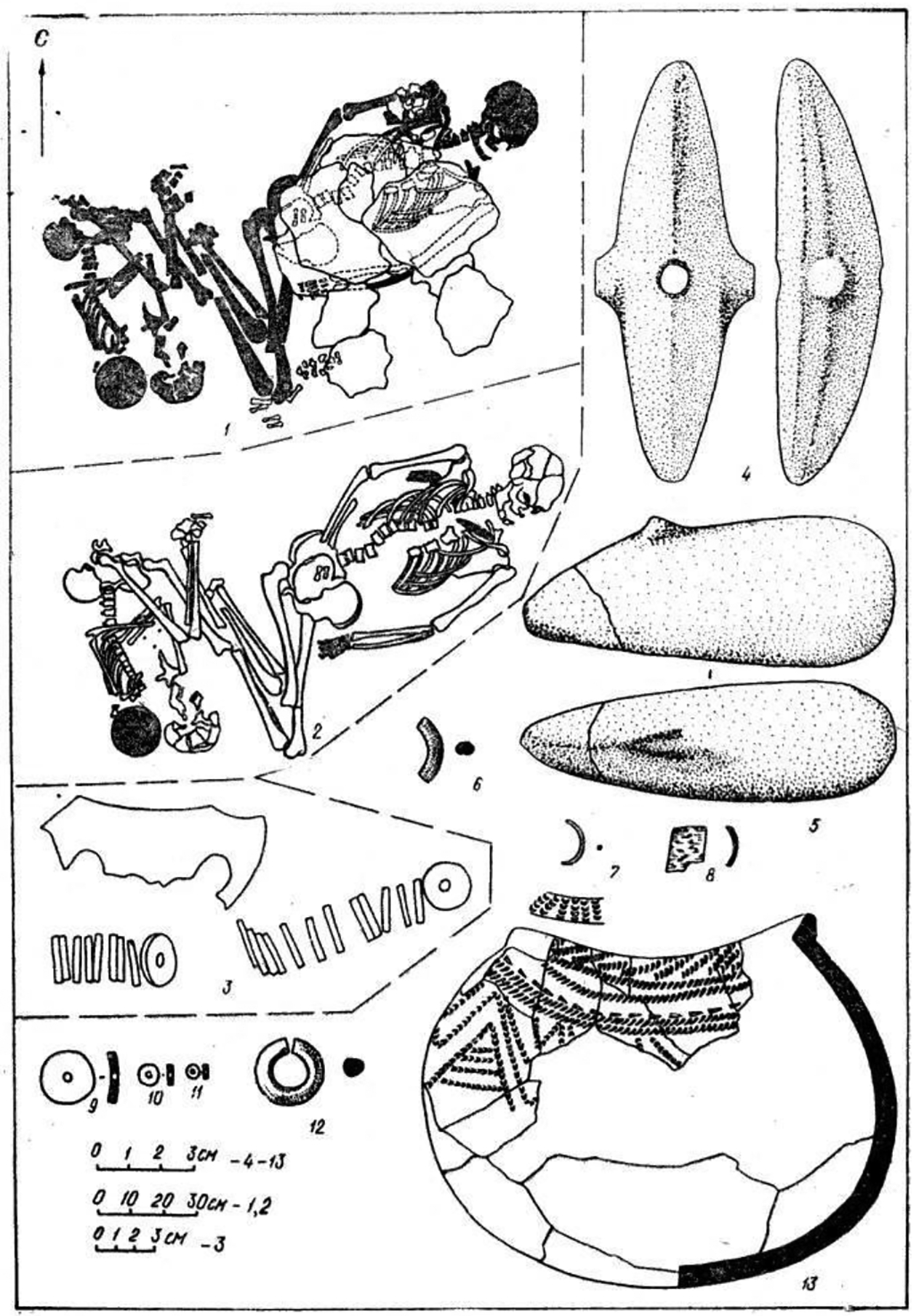
Mace chief grave, Khvalynsk I:108. *1*, skeletons 107–110 with flat stones covering grave. *2*, mace-chief 108 with female 110 in white, maces on chest of 108. *3*, *Unio* shell beads & fragment of a carved bone plaque found with 110. *4,5* maces found with 108. *6*, *12*, stone rings of gray stone, with 108. *7*, copper ring under skull of child 109. *8*, bone ornament fragment. *9–11*, shell beads with 108. *13*, ceramic cup 11 cm high found with female 110. After Agapov et al. 1990, Figure 21.

The other mace grave at Khvalynsk I was I:108 (Figure 12). This grave held two maces, one whole and one broken (Figure 1: top, middle right; Figure 12: 4,5). They were on the upper chest of a male described as ‘young adult’, age not given (Agapov et al. 1990: 44). His skeleton is now unfortunately lost, like I: 57. Male I:108 was on his back with tightly raised knees, fallen to one side, with red ochre around his pelvis. The partial bones of three individuals were at his feet—a ‘young’ female contracted on her right side (I:110), the skull of a child aged 3–5 (I:107), and the skull and long bones of another child (I:109), age not recorded. Near the skull of this child (I:109) were *Unio* shell beads stained with red ochre, and under the skull was a fragment of a copper ring. The partial skull of a sheep-goat was found under the skull of female 110, and a miniature ceramic cup 11 cm high was placed with her (Figure 12:3,13). Male I:108 had a polished stone ring and wore strands of shell beads (Figure 12:9–12). His was the only mace-grave that did not contain a bird-bone tube, a copper object, or an animal sacrifice, although an animal sacrifice (110) and a copper item (109) were found in the same burial cluster. The broken mace buried with him was missing its narrow end, the end that was mounted in a haft (Figure 1: top; Figure 12). The matching broken end piece was found 2m to the north in grave I:104 (see Figure 14 for location), reportedly in a rodent burrow in the floor of the grave (Agapov et al. 1990:44). Grave I:104 contained an adult female aged 25–35, wearing copper and shell ornaments, lying on top of the mace fragment, with the curated skull fragments and long bones of a child 3–7 years old (I:106) beside her.

The male in I:108 curated the broken mace made of yellow-brown stone (Figure 1:top and 12:5) but also acquired a new, unbroken mace of a different, cruciform type, made of dark grey stone (Figure 1: middle right and 12:4). The curation of the broken mace, its replacement by a new whole mace, and the possibility that the broken piece was intentionally placed in the adult woman’s grave vividly attest to the power infused into these objects by the people at Khvalynsk. Discussing religion at Çatalhöyük, Hodder (2014: 22) observed that the Neolithic population there seems to have regarded some material objects, including houses, as imbued with a vital, living force that empowered them with spiritual agency. The context of the two maces in Khvalynsk grave I: 108 suggests that these iconic symbols were regarded as possessing vitality and agency in a similar way, perhaps related to the vital power of their owners.

**Figure 13:**
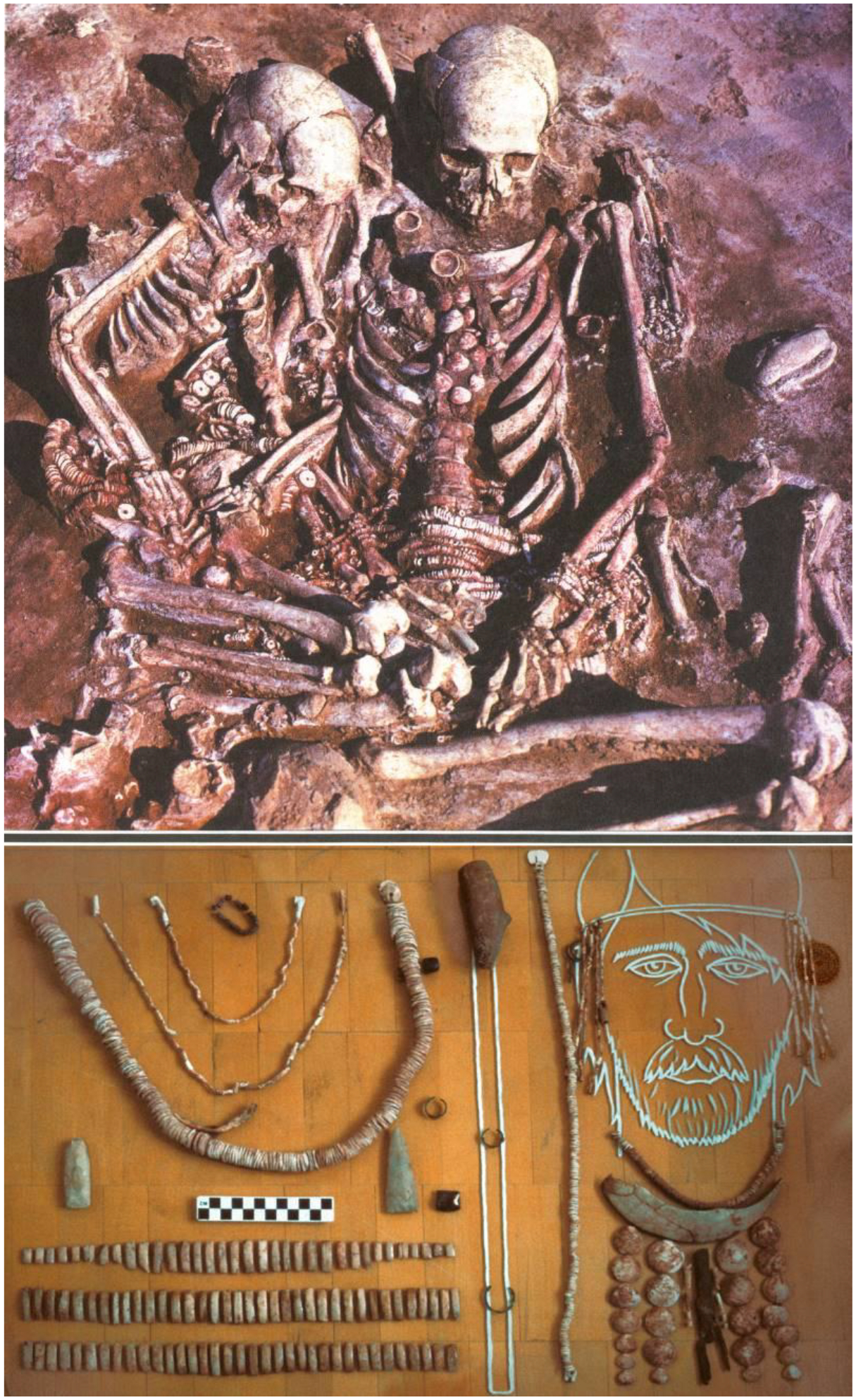
A: Mace chief grave, Khvalynsk II: Grave 25 (left, sister) & 24 (right, brother). Mace, center right; sheep and goat bones, bottom right. B: Artifacts with brother include an eared mace (top center), belts of *Unio* shell beads (top left), belts of 194 beaver incisors (bottom left), two stone adzes (left center), a boar’s tusk pendant, *Glycemeris* shell pendants (below face), copper beads, rings, bands, and a spiral ornament (right), and a flint bifacial projectile point (not shown). A. *in situ* photo by I. Vasiliev, color correction by D. Agapov and N. Agapova, copyright A. Agapov 2010, used with permission. B. Exhibit dated 1992, Institute for the History and Archaeology of the Volga (IHAV), Samara, Russia. Photo by D. Brown

The third mace grave was the only grave at Khvalynsk II that contained copper *and* animal sacrifices *and* an adult male, like I:57 at Khvalynsk I. Although grave II:12 contained more pieces of copper, grave II:24 was the richest grave at Khvalynsk, defined by the most diverse collection of grave gifts, including an eared stone mace (Figure 13). This is the only Khvalynsk mace-chief that is preserved, so can be analyzed using modern methods.

The male aged 20–25 (II:24) was buried with a female aged 8–9 (II: 25), his sister, with the partial remains of three other individuals at their feet: a male 16–19 years old (a 3^rd^-degree relative), and two infants (not analyzed for aDNA). Whole genome analysis revealed that 24 & 25 were brother and sister, although born at least 10 years apart. She wore many decorative belts of riverine *Unio* shell beads. Copper rings and beads were found only on the male, and beside him was an ‘eared’ mace like the broken one in Grave 108, and 14 bones from a sheep *and* a goat (Bogatkina 2010: 400). He was buried wearing multiple mid-body belts of *Unio* shell beads, multiple mid-body belts made of 194 beaver incisors (or a shirt covered with beaver incisors?); a boar’s tusk chest pendant; fossil *Glycemeris* shell pendants (a marine shell also used at Varna for ornaments); a tubular bird bone; and 14 copper ornaments consisting of beads, rings, a spiral ornament or coil of wire (Figure 8:5), and bands that might have been wrapped around wooden shafts (Figure 9). He also had two lumps of melted copper, perhaps signs of metal craft working, or perhaps copper trade ingots. The male’s Y-chromosome haplogroup was Q1a1b, a Siberian, northern haplogroup; for example, almost all the males at Murzikha, a contemporary cemetery in the forest zone, were Q1a (Figure 2). His and his sister’s MtDNA haplogroup was U2e1b, also found in Mesolithic individuals in Latvia and Siberia, so again a northern lineage. His paternal ancestry contrasted with the paternal ancestry of most of the males at Khvalynsk II.

Maces at Khvalynsk represented a specific and unique status reserved for the two adult males who belonged to both the copper and animal-sacrifice depositional groups simultaneously, and for one other adult male who was buried in a complex group grave that contained copper and an animal sacrifice deposited with other individuals in the group. The intersection between adult male + copper user + sacrifice receiver was marked using polished stone maces by the ancient population itself. The repetition of this prehistoric act twice, in the only two graves at Khvalynsk where copper + animal sacrifice + adult male clearly coincided, and perhaps a third time if we relax the rules a little, suggests that the copper-receivers and the sacrifice-receivers had real social salience. We do not know what these groups represented, whether they had broad Dumézilian social functions as we have suggested (priest vs. warrior/trader), or if they symbolized something more specific. But whatever they meant, they were recognized by the Khvalynsk people—and their intersection was marked with maces. The maces therefore perhaps symbolized generally the integration of socially separate groups through the medium of a single adult male who symbolically balanced their interests and facilitated their union—a chief.

## Maces and head trauma at other sites

**Figure 14:**
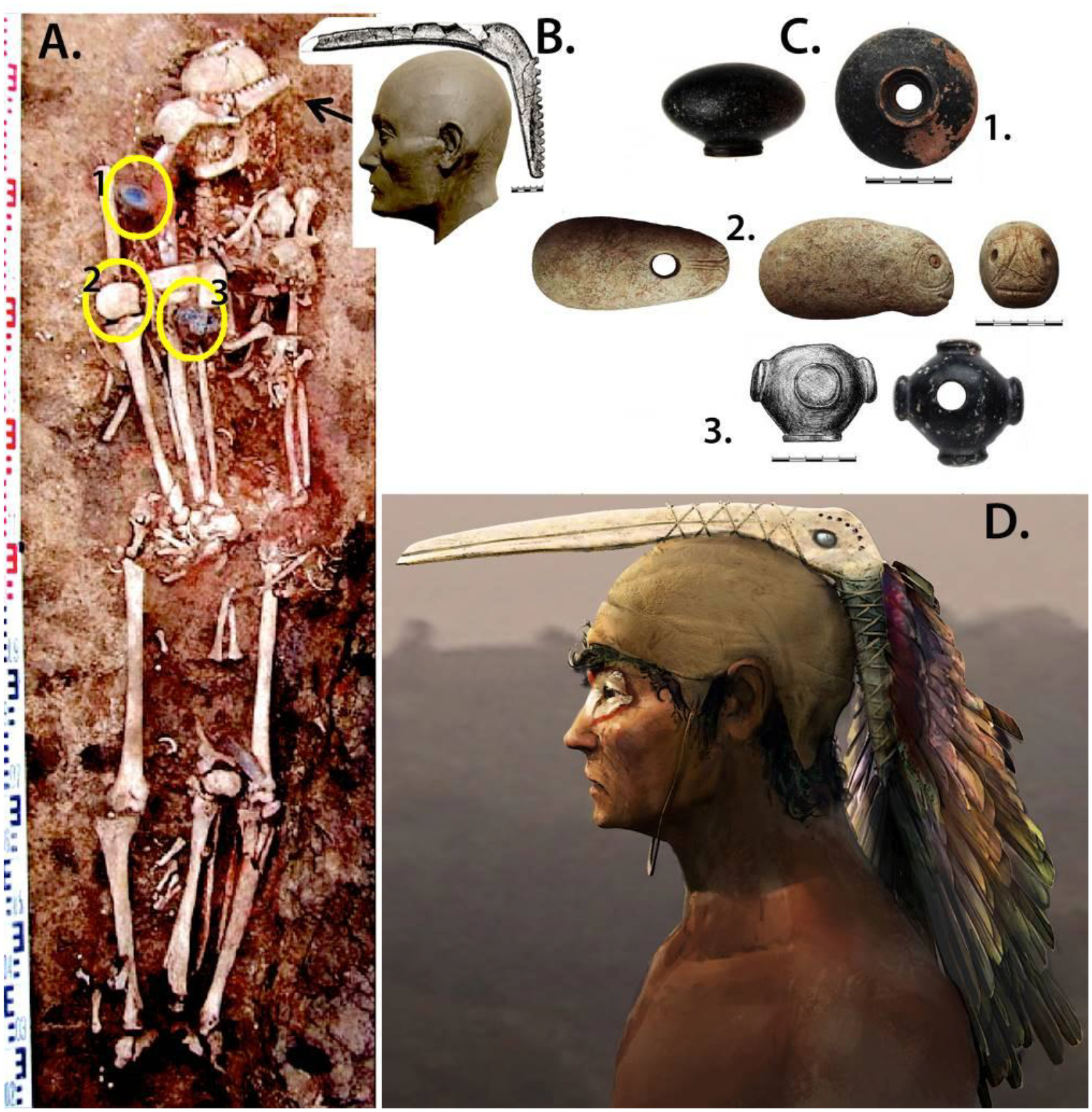
Ekaterinovka Mys grave 45. A. Photograph of grave 45 with three mace heads circled. Two tibia bones from two other individuals rest against his right arm and between his own tibias. Two severed hands of two individuals rest on his left hip. A domesticated goat kid rests on his left arm. B. Reconstruction of the head and face of grave 45, with line drawing of the carved antler bird head. C. Three mace-heads from grave 45, found on his right arm. D. Artist’s rendition of the young male with the antler bird arranged as if it were the crest of a hat. A,B, and C are after Korolev et al. 2018, Figures 2, 4, 7, 9, 10, & 12. D is by Russel Story.

Polished stone mace-heads were buried in Eneolithic graves in the Dnieper steppes (Mariupol) and the North Caucasus steppes (Merekli-Tekeb), and they later appeared as imports in Varna-era agricultural towns in the Danube valley and in Cucuteni A/Tripol’ye B1 towns in the eastern Carpathian piedmont. It was once argued that they were products of the Varna-era agricultural towns that diffused eastward into the steppes (Govedarica and Kaiser 1996), but later studies claimed that they are more numerous in the steppes, with older dates (Dergachev 2007). In relation to these debates, the four maces at Khvalynsk are important because of their well-dated archaeological contexts. But they were not the earliest dated maces in the region. That distinction belongs to the polished stone mace-heads from the Ekaterinovka Mys cemetery (Figure 14).

Ekaterinovka Mys (‘Mys’ means ‘peninsula’) is a Volga riverside Eneolithic cemetery of 100+ graves 150 km north of Khvalynsk, below the big Volga loop known as the ‘Samarskaya Luka’, or the Samara bow, near the city of Samara. Here the Volga makes a 160-km loop around a limestone mountain that rises more than 300m above the river, a prominent feature that coincides broadly with the ecological border between the steppe and forest-steppe vegetation zones. Ekaterinovka Mys was situated at the southern or steppe end of the Luka, at the transition to the steppes.

The radiocarbon dates on human bone are skewed too old by FRE, but we also have dates on animal bones or teeth unaffected by reservoir effects. In Table 4 we present five dates from Ekaterinovka Mys from terrestrial animals, including a goat, a sheep, and a beaver. Like beavers at other sites (Wood et al. 2013), the beaver incisor dated here has stable isotopes indicating a terrestrial diet (δ13C −20.7, δ15N 6.7). The human from grave 45, buried with the dated goat kid, yielded a FRE-skewed radiocarbon date 800 years older (5311–5218 calBCE/6280±25BP/ PSUAMS-2882). An organic residue from a potsherd gave a date (Korolev et al. 2019:29) like three animal bone/tooth dates; one date on a sheep tooth was somewhat older. The average midline for the five dates was 4618 BCE, about 200 years older than the average midline from terrestrial animals at Khvalynsk, 4400 BCE. The sheep and goat dates are among the oldest dates for domesticated sheep and goats in the Volga steppes (excluding anomalous dates on organic residues) (Vybornov et al. 2018). But the male in grave 45 was buried with much more than a domesticated goat. He had three maces.

Figure 14:C illustrates the three drilled polished stone mace-heads placed on the right arm of the man in grave 45, reproduced from Korolev et al. (2018). They are surprisingly diverse in shapes and colors, made of different stones: one ovoid, one four-lobed, and one (Figure 14:2) zoomorphic, probably meant to resemble a fish head (more like a catfish than any other Volga fish). No copper was found in this or any other grave at Ekaterinovka Mys, a strong contrast to Khvalynsk (Korolev et al. 2018: 287); and very few domesticated animals were sacrificed during the funerals at Ekaterinovka Mys, another contrast to Khvalynsk. But the young male (aged 20–25) in grave 45 was provided with a domesticated goat kid deposited on his left arm (see goat skull on male humerus, Figure 14:A). Also, in addition to the three maces deposited on his right arm, the young male had two severed hands from two different people placed on his left hip; and two severed lower legs (from the knee down, including tibias and some foot bones) of two people, probably the same two victims. One severed lower leg was on the young male’s right side with the three mace-heads; the other was placed between his lower legs (Figure 14:A). These severed body parts from two people appear to be war trophies. It is possible that two of the three mace-heads in grave 45 also were war trophies and were associated with the two victims.

A piece of elk antler carved in the shape of a long-beaked bird lay across the head and face of grave 45 (Figure 14:A & B). It had use-worn, polished serrated notches on the bird’s ‘neck’, as if used for the attachment of decorative suspensions, like bundles of feathers. Figure 14:B shows the carved antler bird arranged over the reconstructed head and face of the young male in grave 45 as if it were the crest of a feathered hat (images revised from Korolev et al. 2018). Figure 14:D shows an artist’s impression of how the hat might have been worn, created by free-lance artist Russell Story, who creates imagery for Industrial Light and Magic within LucasFilms. The species of long-beaked bird represented in the carving (13:B) is unknown, but here is represented as a glossy ibis (*Plegadis falcinellus*) because this is the longest-beaked Volga bird, and the carved beak looks longer than a crane or heron beak. In the artist’s rendition (12:D), the feather mantle is made of glossy ibis wing feathers, and the paint around the man’s eyes uses white and red colors from glossy ibis eye-patch feather colors. In the lower Volga marshes, near the Caspian Sea, glossy ibises gather each summer to feed and breed before migrating south for the winter. If the carved antler bird *was* meant to represent a glossy ibis, it is interesting that Ekaterinovka Mys is hundreds of kilometers north of the ibis breeding zone on the lower Volga, so an ibis head-dress might suggest a southern provenance, possibly a gift from the south. The annual migrations of glossy ibis could be an animal-world metaphor for the north-south movements of human groups that are reflected in the heterogeneous genes and cranio-facial types at Khvalynsk and to a much lesser extent at Ekaterinovka Mys.

The Eneolithic maces at Khvalynsk, Ekaterinovka Mys, and other steppe sites (Dergachev 2007) must be understood not only as aesthetically attractive symbols, but also as status weapons that threatened violence. Unlike a knife or axe, a mace has no non-violent function; it is designed to break skulls. Those skulls might be of large fish (Volga catfish could weigh more than 300kg and probably were killed by blows to the head) or sacrificial animals. But grave 45 at Ekaterinovka Mys shows that inter-human violence also was associated with mace-holders in the centuries before Khvalynsk.

Increased violence is not evident in the few studies of pathologies in Eneolithic skeletons in the Volga steppes. Khokhlov did not feel that violent trauma was significant in the Khvalynsk population. But this is largely because an odd and puzzling trait on many Eneolithic skulls that would normally be interpreted as indicating violent blows to the head has instead been interpreted by three different experts as ritual, not connected with violent blows but rather with intentional scraping or gouging of the skull.

These oval, saucer-shaped depressions in the parietal bone are called “ritual trepanations” by Khokhlov, who counted 10 cases at Khvalynsk II, all adults (2010:418–419), and nine cases at Ekaterinovka Mys, including the male in grave 45. The gouged-but-not-hit skulls at Khvalynsk II were noticed also by Murphy in her internal report for the Samara Valley Project, but she did not describe them in print (Murphy 2016). In the cases at Khvalynsk and Ekaterinovka Mys, the outer layer of skull bone was scraped away, making small ovoid depressions that did not penetrate through the inner layer of bone. Gresky et al. (2016) documented a similar but more extreme skull modification ritual in the North Caucasus steppes at Progress-2 and Vonyuchka-1 (also known as Konstantinovskii-1), in which full trepanations were conducted, with penetration through both the inner and outer layers of bone, on people who showed no sign of skull trauma or injury. These features did not exhibit the radiating cracks or crushed edges that accompany a violent blow, but were created for unknown reasons, perhaps (by analogy with actual trepanations) to relieve other sources of head pain. They represent a confusing factor in attempting to evaluate the level of inter-personal violence in the Eneolithic, because they look very much like head trauma and might disguise trauma, but they are not themselves the result of violence. Gresky et al. (2016) counted these features on fully 10% of the Eneolithic skulls they examined from graves between the North Caucasus steppes and the lower Don. They document a ritual that was shared across the Volga-Don-Caucasus steppes among Eneolithic people who also exhibited similar genetic ancestries and similar styles of polished stone maces.

The integrative, inter-group bridging function suggested here for the copper-rich mace-chiefs at Khvalynsk could have been a peace-making reaction to the violent trophy-taking of the previous century, illustrated at the Ekaterinovka Mys cemetery. An ethnohistoric analogy might be the creation of the League of the Iroquois in what is now New York state. The League was a peacemaking alliance between five powerful tribes that had previously experienced chronic inter-tribal warfare (Birch and Hart 2018:18). A document-based interpretation of the League (Starna 2008) suggests that it functioned to sustain and facilitate access among the five tribes to novel and highly desirable European trade goods. These are paralleled on the Volga by copper objects introduced by the Danubian centers of what Chernykh (1992, 2008) named the ‘Carpatho-Balkan Metallurgical Province’. Iroquoian belief systems present a different picture of the founding of the League, describing it as a religious awakening inspired by a culture hero (known as the Peacemaker) who introduced new, integrative rituals. Similarly, at Khvalynsk we witness the appearance of new sacrificial rituals in which domesticated mammals were a mandatory medium for the funeral ceremonies of a minority that distributed funeral feasts to hundreds of mourners. These funeral gatherings, and the integrative symbolism of the chiefs who oversaw them, could be signals of a broader social integration or inter-regional confederation, partially inspired by the desire to reduce conflict and facilitate trade in novel copper artifacts.

## Funeral rituals in the Volga-Caucasus Eneolithic

**Figure 15:**
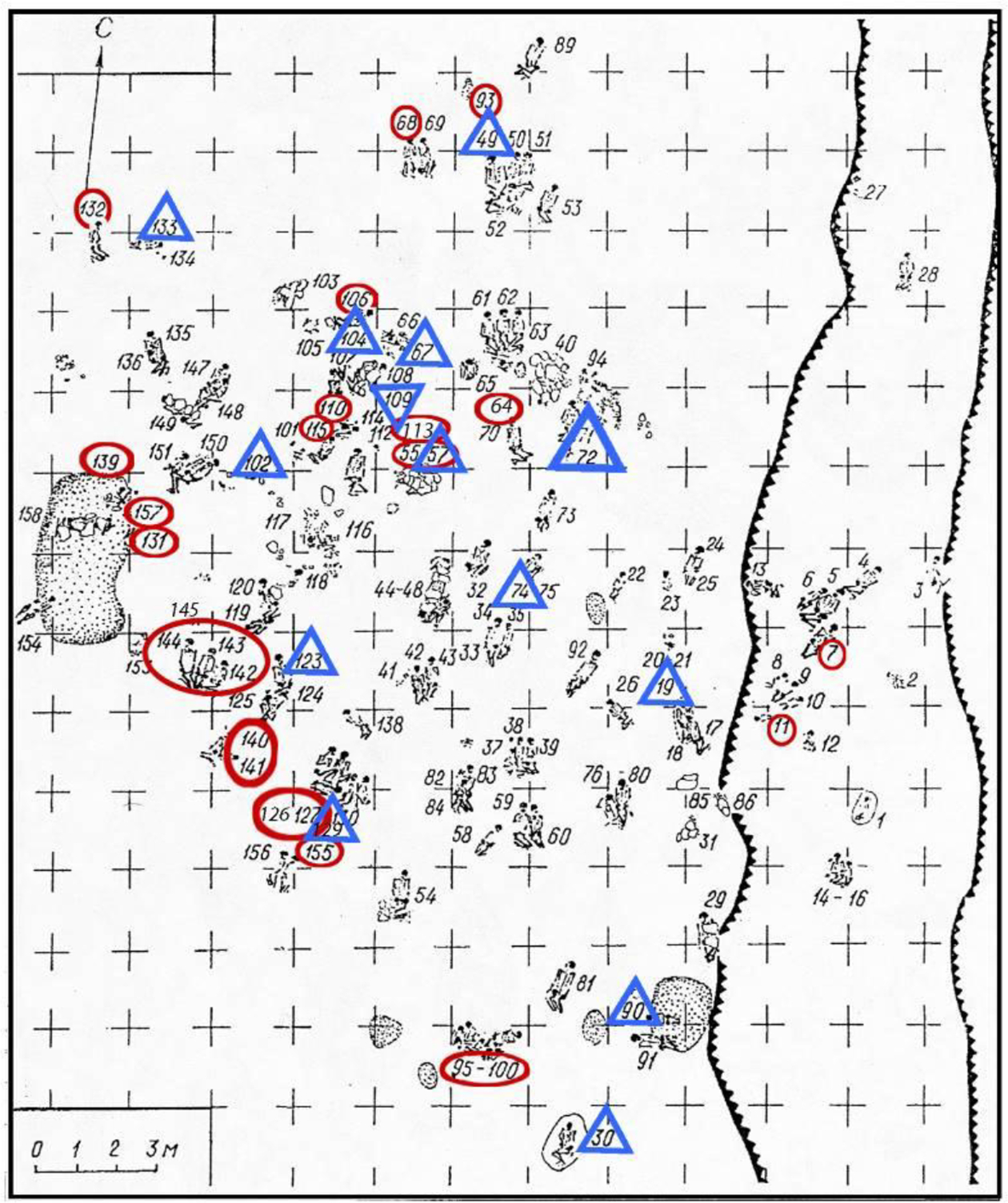
Plan of Khvalynsk I. Red circles = animal sacrifices; Blue triangles = copper finds; Stippled areas = surface sacrificial deposits in red ochre; Serrated lines = Volga River bank in 1977 and 1979. Arrow with ‘C’ denotes north. Graves 90 & 91 are illustrated in Figure 9, graves 147–149 in Figure 5. Total area explored was larger; see shaded area in Figure 4. After Vasiliev 2003.

The spatial arrangement of the graves at Khvalynsk I was described in maps, plans, and figures in Agapov et al. (1990). Small errors on the cemetery plan were corrected by Vasiliev (2003: Figure 1), the basis for Figure 15. The only published plan of Khvalynsk II is in the article on copper metallurgy by D. Agapov (2010: Fig.5) in the Khvalynsk monograph (S. Agapov 2010), which is the basis for Figure 17.

**Figure 16.**
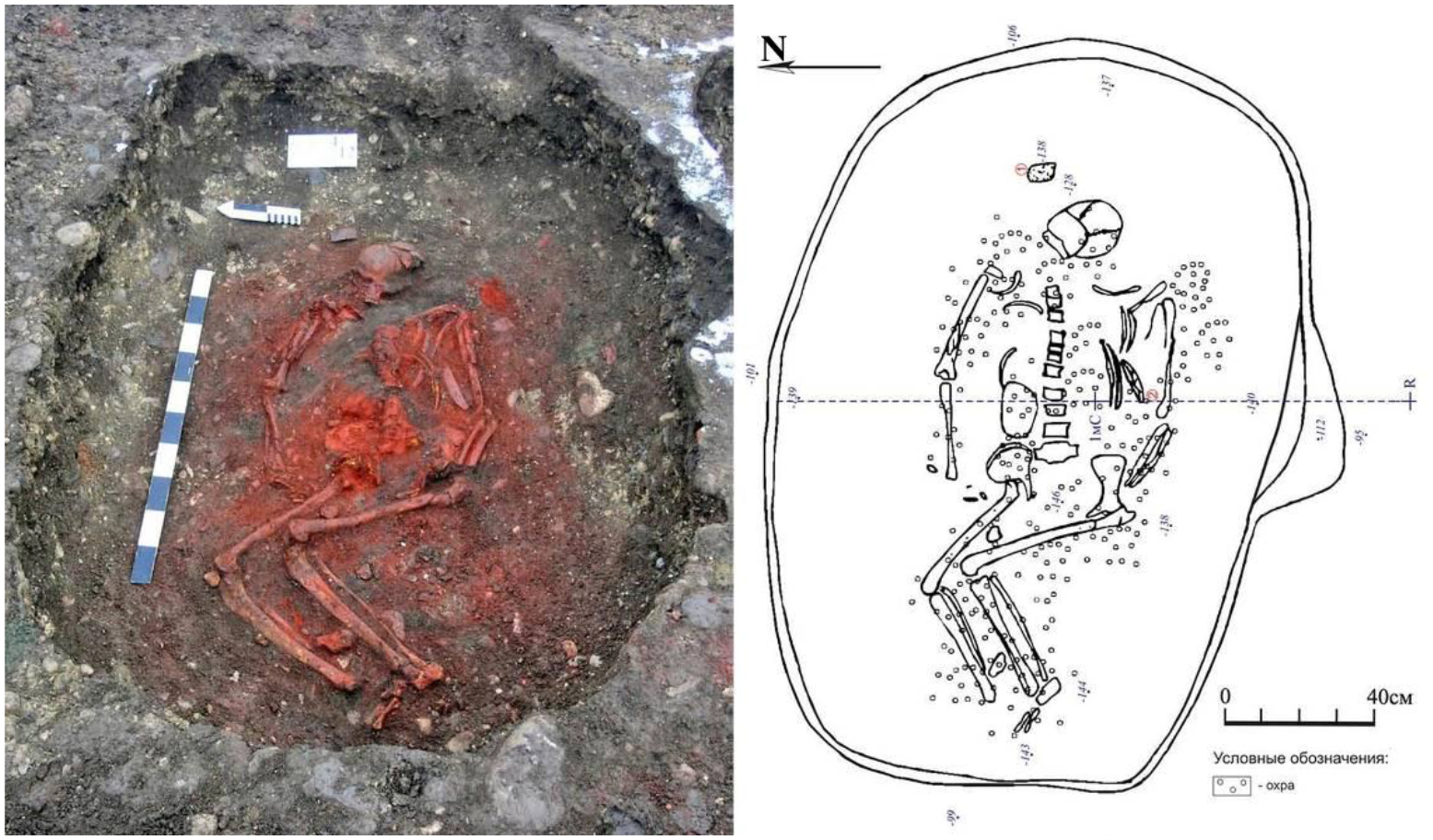
Progress-2, near Nalchik, Russia. Kurgan 4, grave 12. Male 25–29 with ‘ritual trepanation’. Flint blade 13.6cm long on ribs, right; ceramic sherd above skull, left. Ceramic sherd was from same vessel as sherd in grave 9, partly visible in photo upper right beside grave 12 under same mound; grave 9 was sampled for aDNA. Grave 12 was dated 4232–4048 (95%) (5305±25 MAMS-11211); grave 9 dated 4233–4047 calBCE (5304±25 BP, MAMS-11210). After Korenevskii et al. 2019, used with permission.

The arrangement of graves within Khvalynsk I and II did not follow a consistent rule. The cemetery plan does not reveal straight rows of graves, or clearly separated groups. The linear distance between graves was highly variable, so some graves were crowded near each other, while other graves were scattered apart. Genetic analysis of family relationships at Khvalynsk II (Figure 16) raised the possibility that the individuals belonging to the largest family (designated the Yellow family) were buried in what appears to be an east-west row; but this pattern, if we can call it that, was not maintained. Graves of other families clustered around the ‘Yellow’ row in no apparent pattern, creating a cemetery plan in which no rows or alignments could be perceived. Rows of graves were more apparent at Ekaterinovka Mys, 150 km north, where the rows were aligned NW-SE with heads oriented NE. At Khvalynsk I, graves were aligned toward the N, NW, or NE, most to the NE. At Khvalynsk II, the same N-NE orientation was standard, although the richest grave, the mace chief (II:24), was oriented SE.

The standard body pose at Khvalynsk was highly distinctive—on the back with tightly raised knees—and would later be characteristic of early Yamnaya graves. About 55% of the individuals at Khvalynsk I and II were arranged in this pose, which predominated among both men and women (Agapov et al. 1990: 57); another 10% were buried in a contracted position on one side; and less than 10% were buried in a ‘sitting’ position with the upper body slightly raised and the back curved, originally posed with elbows down, and the knees raised. The sitting position occurred as a minority pose also at other regional cemeteries, including Ekaterinovka Mys. Most of the other individuals were secondary or partial skeletons, often just the skull and a few other bones, in which pose could not be determined.

The supine-with-raised knees burial pose was a defining steppe-zone Volga funeral custom, seen also at Khlopkov Bugor and in Eneolithic graves at Engels and on the lower Volga (Dremov and Yudin 1992). A different position—supine with legs extended straight—was standard in graves at Ekaterinovka Mys (Figure 14) and S’yezzh’e at the northern edge of the steppes, and in Neolithic cemeteries on the Dnieper.

In the North Caucasus steppes at older Eneolithic cemeteries such as Nalchik (4840–4820 BCE, GrA-24442, 5910 ± 45 BP), the flexed pose, contracted on the side, was used for most individuals, but even here a few individuals were buried on the back with raised knees. When the first small earthen mounds, or kurgans, began to appear in the North Caucasus steppes during the Eneolithic, after 4500 BCE, they were erected over graves in which the deceased was positioned supine with raised knees, usually oriented to the east (Korenevskii 2012; 2016). The Eneolithic individuals at Progress-2 and Vonyuchka in the North Caucasus steppes who had genetic ancestry similar to Khvalynsk were buried in the Khvalynsk position, in graves intensely colored with red ochre, beneath small (less than 1m high, ca. 15m diameter) earthen mounds (Figure 16). These mounds were among the oldest kurgans in the Pontic-Caspian steppes (Korenevskii 2012); the other region where small kurgans appeared this early was in the steppes north of the Danube delta, as at Suvorovo (Anthony 2007:253), again at a cultural, economic, and genetic border (Figure 2). Although they were small compared to later Yamnaya kurgans, the Eneolithic kurgans in the upper Tersek steppes east of the Svobodnoe-Meshoko agriculturalists perhaps were a boundary-marking practice that emerged during the late fifth millennium BCE. This was a millennium before the Yamnaya culture made the kurgan type of funeral monument universal across the Pontic-Caspian steppes.

The Skelya and Sredni Stog cultures in Ukraine, contemporary with Khvalynsk, also used the supine-with-raised-knee posture, unlike the supine-extended burial pose in the Dnieper Neolithic cemeteries (Telegin and Potekhina 1987). Sredni Stog individuals also had genetic ancestry more like Khvalynsk and Progress-2 than the Dnieper Neolithic ancestry type (see below). Sredni Stog lithics also were similar to Khvalynsk, particularly the use of large lanceolate projectile points and long unifacial lamellar flint blades. Sredni Stog pottery was tempered with crushed shell, like Khvalynsk pottery, and unlike the Neolithic pottery of the Dnieper valley, where sand or mineral temper had been used. The high percentage of horse bones, averaging more than 50% of all animal bones in Dnieper-valley Sredni Stog sites, was consistent with the high importance of horses in the diets of many fifth- and fourth-millennium BCE Eneolithic settlements in the Volga and Don valleys (Anthony and Brown 2011). Many traits indicate ‘eastern’ influences on Sredni Stog material culture, economy, and genetic ancestry, and the supine-with-raised-knee burial pose is one of these.

At Khvalynsk I about 30% of the graves were single, about 20% held a pair of individuals (eg, I: 68 & 69), about 20% had three individuals (I: 61–63), and the most complex graves contained individuals buried with parts (often skull parts) of multiple other individuals and with additional individuals at their feet (I: 107–110), or individuals arranged in layers with multiple adults laid out over other adults who had additional individuals at their feet (I: 126–130). All three mace graves were complex multiple interments (I;55–57, I: 107–110, and II: 23–25, 23a, 23b). At Khvalynsk I, the two mace graves (I: 57 and I: 108) were only two meters apart in the most crowded part of the cemetery. Graves with animal sacrifices seem to be arranged on the periphery of the cemetery and copper-users seem concentrated more in the center, but they overlap spatially considerably. The spatial layout of Khvalynsk II is considered below with the DNA evidence.

Pestrikova (Pestrikova and Agapov 2010) attempted to discern sub-groups within Khvalynsk I based on the depth of the grave, the age and sex of the individual, the use of red ochre, special artifact types such as bird-bone tubes, and other traits. She suggested five principal social groups or categories, with the two highest-prestige categories being interpreted as ‘leaders’ and ‘shamans’. This is not so different from the suggestion here that animal sacrifices designated a sacral minority group and copper ornaments designated a warrior/trader minority group. Pestrikova did not consider animal sacrifices in her analysis, and her suggested five groups were not borne out at Khvalynsk II, where the traits that defined her Khvalynsk I categories were mixed. This essay presents a hypothesis about four social categories at Khvalynsk based on animal sacrifices, copper items, maces, and the residual majority without them. These groups seem to apply equally well to Khvalynsk I and II. Other ways of looking at this cemetery would be possible and should be pursued in the future.

## Khvalynsk genetic relationships and cranio-facial types

Genome-wide data from more than 50 individuals from three Eneolithic cemeteries between Samara and Saratov on the Volga are under study in a parallel work. The cemeteries are Ekaterinovka Mys, Khvalynsk, and Khlopkov Bugor, mentioned above and mapped in Figure 2. Here we summarize genetic results relevant to Khvalynsk from the comprehensive report that will be published elsewhere (for questions about the genetic analysis and early access, please write to I. Olalde and D. Reich).

Until now, three individuals from Khvalynsk II (II:1, II:12, II:17) were the only published whole genomes of Eneolithic individuals from the Volga-Ural steppes. First referenced under the generic label Steppe Eneolithic in a report that did not mention Khvalynsk (Mathieson et al. 2015), they were cited briefly three years later in Damgaard et al (2018), and their admixture components were illustrated in Wang et al (2018: Figure 2c). They were first analyzed in detail in a Supplementary Information document attached to a primary report that again did not refer to Khvalynsk (Narasimhan et al. 2019: SI 230–231). For colleagues in genetics they were easily referenced in shared online databases, but for colleagues in archaeology they remain largely unknown. What follows includes these three, but also uses preliminary information from the larger set of individuals now under study by Anthony, Reich, Olalde, and others.

### Relatives and families from ancient human DNA

Most of the 158 individuals from Khvalynsk I were lost in a flood. Five individuals were preserved and passed aDNA screening, and none were related (see Table 6 for their sex-linked haplogroups). This discussion is about family relationships at Khvalynsk II.

At Khvalynsk II 43 individuals were recovered, 77% of them males, a sex ratio like later Yamnaya kurgans in the middle Volga steppes, and unlike Khvalynsk I, where the sexes were equally represented. After screening, 26 individuals had whole-genome data sufficient to analyze family relationships at the level of 1^st^ degree (parent, sibling, child), 2^nd^ degree (grandparents/grandchildren, uncles/aunts, nephews/nieces, half-siblings), or 3^rd^ degree (first cousins, great-grandparents, great uncles/aunts).

Of the 26, 18 (70%) were related to at least one other individual and 17 of the 18 (95%) were males (Figure 18). The only female related to another individual (II: 25) was a 9-year-old girl buried with her older brother, the mace chief (II: 24). The other five females at Khvalynsk II that passed screening were unrelated to the males or to each other, within three degrees. Assuming that they were wives of the men, cross-cousin marriage, a type ascribed to Proto-Indo-European speakers by Benveniste (Mallory and Adams 2006: 212–14), is disproved for the Khvalynsk II population, since no adult female was first cousin to any man. Moreover, no mother-son or father-daughter relationships were detected at Khvalynsk II; all mothers, sisters (with one exception), and daughters were buried elsewhere. We can identify one such female relative: a grandmother or great-aunt of the Yellow-family male in II: 4 was buried 130 km downstream at Khlopkov Bugor (KB7). The absence of female relatives at Khvalynsk II was unlike the older cemetery at Ekaterinovka Mys, where three mother-son relationships were recognized in the cemetery population of about 100. In contrast, Khvalynsk II contained three father-son pairs, and brother’s sons (II:22,27) were buried near their paternal uncles (II:12, 13). Patrilineal family relationships connected 70% of the individuals analyzed at Khvalynsk II, which might have been reserved for a paternally related male sodality, with some unrelated females and immatures. In this hypothetical sodality males died at a younger average age than the males in Khvalynsk I (Khokhlov 2010). As noted above, 70–80% of the individuals in Yamnaya kurgans were adult males, as at Khvalynsk II, suggesting that Yamnaya funeral customs could have evolved from the conventions of an Eneolithic male sodality rather than a “culture”.

**Figure 17:**
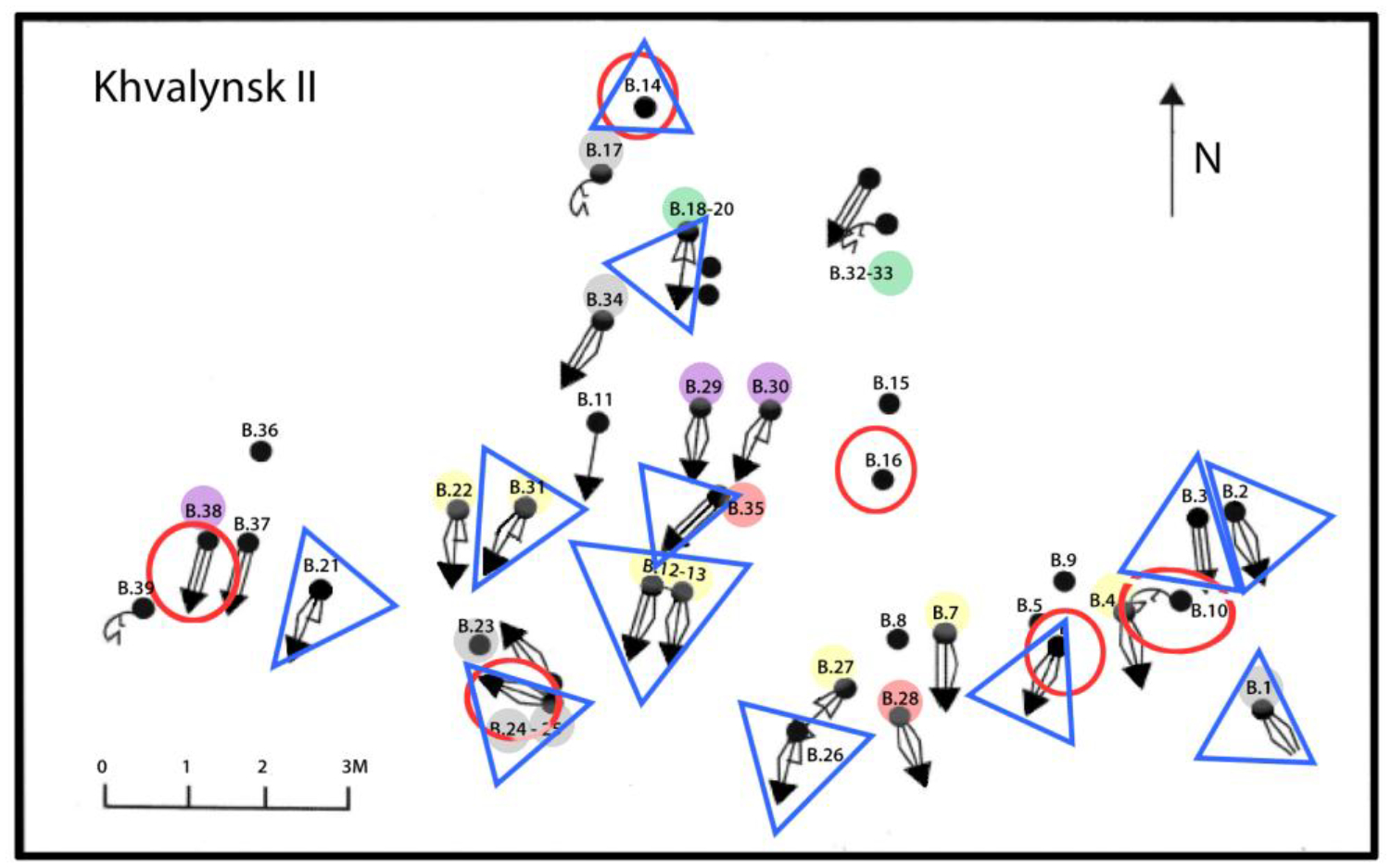
Plan of Khvalynsk II with family relationships indicated by color superimposed on copper finds (blue triangle) and animal sacrifices (red circle). Icons with straight lines were supine with raised knees; icons with curved lines were half-sitting with raised knees; black circles were isolated skulls. Related individuals are color coded with six colors for six families. Table 5 describes the relationships. Adapted from D. Agapov 2010: figure 5.

The 18 related individuals can be grouped into six families of at least two members. The families are given colors in Table 5. The plan of the cemetery in Figure 17 uses the Table 5 color code to identify graves belonging to each of the six families. Their spatial patterning is discussed first.

The spatial arrangement of families at Khvalynsk II shows some patterns (Figure 17). The Yellow family graves were arranged in an east-west line, heads to the N or NE. They are the only family group that shows such spatial coherence. The Grey family graves (II:1, 17, 34, 24, & 25), were placed north, south, and east of the central Yellow-family line. The Purple family (II: 29, 30, 38) graves were arranged on the northern and western periphery of the Yellow family line. The two members of the Orange family (II: 28, 35) seemed to intrude into the space of the Yellow family, but no other family did so. This makes it seem that the Yellow family line of graves around the brothers II:12 & 13 was established first. After these graves were made, other families arranged their graves around the central Yellow cluster. The rich Grey family brother-sister pair in II:24&25 was buried quite near the Yellow brothers II:12&13, perhaps an intentional spatial expression of proximity in power and status. Similarly, the two mace-chiefs buried at Khvalynsk I were within two meters of each other, perhaps another spatial expression of proximity in status.

Table 5 lists 23 binary family relationships between individuals at Khvalynsk II. The Yellow brothers in graves II:12 and II:13 lie at the center of these relationships, participating in almost half of them (11 binary relationships) as brother, uncle, cousin, or grandfather to other males. The Yellow brother II:12 possessed 80% of the copper found at Khvalynsk, so he was identifiable archaeologically as a central figure, but his genetic centrality was not previously known.

**Figure 18.**
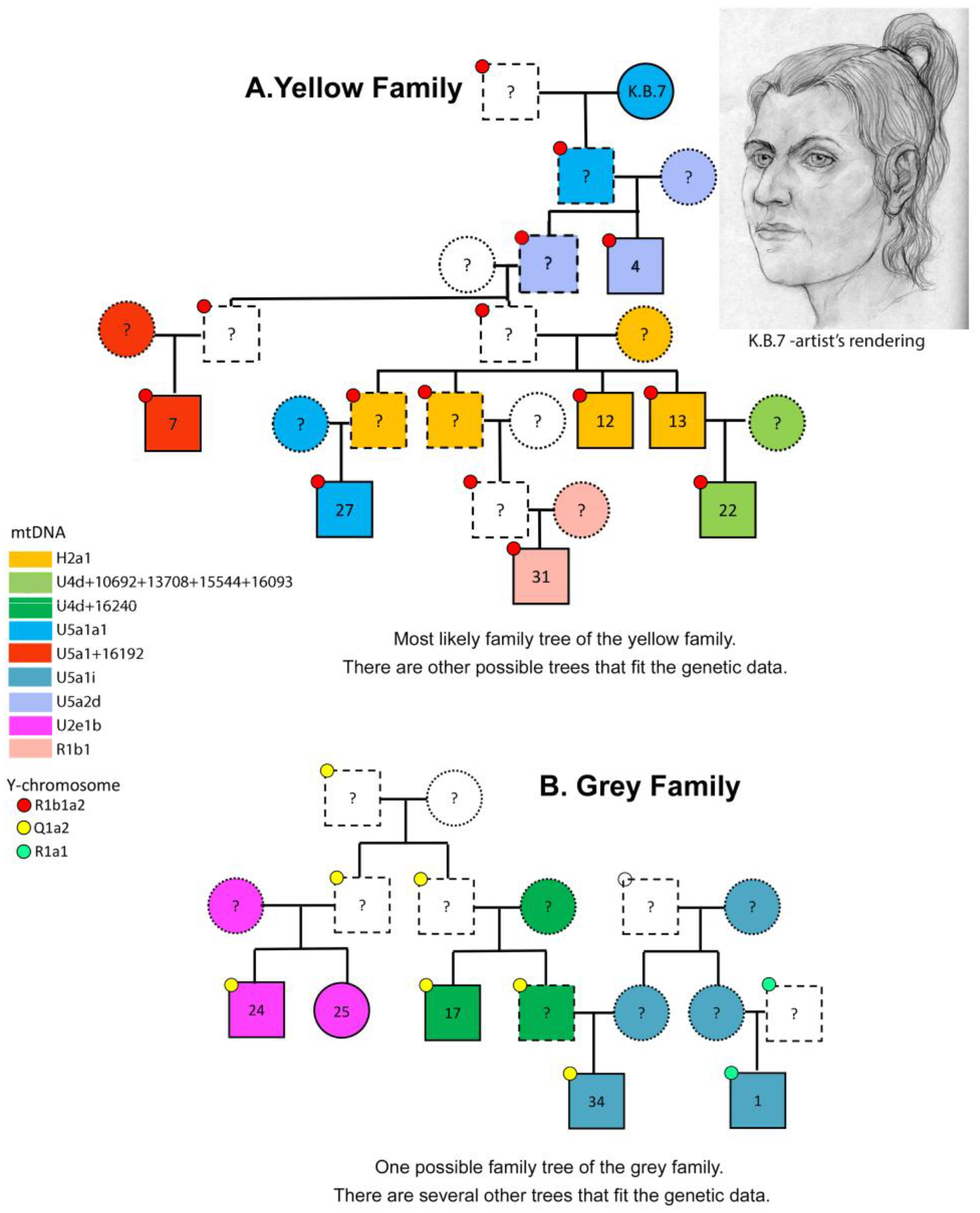
Colors are mtDNA haplogroups, circles in upper left corner are Y-haplogroups according to color key at left. Dashed rectangle=missing male; dotted circle=missing female; solid rectangle=sampled male; solid circle =sampled female. **A.** Most likely family tree of the Yellow family, assuming KB7 is oldest; **B**. one of several equally plausible family trees of the Grey family. KB7 and grave 27 have the same mtDNA haplogroup but they were not related within 3 degrees so any relation was several generations back.

In addition, the Yellow-family male in grave II:4, buried with a bird-bone tube (but without copper or animal sacrifices), was a 2^rd^ degree relative, modeled in Figure 18 as a grandson, of a Yellow family female buried 130 km to the south in grave 7 at Khlopkov Bugor (KB7), a Khvalynsk-culture cemetery of 24 graves near modern Saratov. The absence of copper at Khlopkov Bugor makes it likely that it was older than Khvalynsk, but within the chronological limits of 2^nd^-degree relatives, so no more than two generations older. Since the mtDNA haplogroup of II:4 differed from KB7, the female in KB7 was either his paternal aunt or paternal grandmother. His would then be among the oldest graves at Khvalynsk II. During the brief interval between Khlopkov Bugor and Khvalynsk, perhaps around 4500 BCE, Balkan copper began to flow through exchange relationships in the Volga steppes and was concentrated at Khvalynsk.

The colors in Figure 18 designate sex-linked haplogroups, not families. The mtDNA haplogroup is the main color and the Y haplogroup group is the corner color. The corner colors for Y-haplogroups in Figure 18 are used again in Table 6, which shows all sex-linked haplogroups detected at Khvalynsk I and II. Nine mtDNA haplogroups and four Y haplogroups are listed. R1b-L754 was the most common Y-haplogroup and U5a was the most common mitochondrial group. Five individuals from Khvalynsk I had four mitochondrial haplogroups (T2a, U2e, U4a, U5a), all of which were shared at Khvalynsk II; and two paternal Y-haplogroups, one of which (I2a-L699) was unique, while the other (R1b-L754) was shared at Khvalynsk II. In Table 6, Y-haplogroups are listed with both their alpha-numeric code out to three digits (as in R1b) and their Y-full tree (https://www.yfull.com/tree/) designation (as in R1b-L754). Evolutionary lineages within a Y-haplogroup branch are shown as in R1b-L754 > L389 > V1636, where L754 is basal, L389 is derived from L754, V1636 is derived from L389, and all the SNPs out to V1636 are preserved. The difference between R1b-L754 and R1b-V1636 can be caused by differential preservation of SNPs, so does not indicate different paternal ancestry.

Figure 18:A presents a best-fitting family tree for the Yellow family individuals, limited by making II:4 among the oldest Yellow individuals, and by Y-chromosome and mtDNA haplogroups (Table 6) as well as permissible degrees of relationship (Table 5). Six maternal mtDNA haplogroups were present in the Yellow family (Figure 18:A & Table 6), and one paternal Y-haplogroup (R1b). The oldest modeled Yellow male, II:4, was a 3^rd^-degree relative, probably a paternal great-uncle, of the Yellow brothers II:12&13. If this sequence is correct, then after the brothers II:12&13 were buried, three more Yellow family males (22 and 27, then finally 31) were buried on either side of II:12&13. The Yellow family is modeled as present at Khvalynsk over five generations, although we lack graves from generation two, between II:4 and the II:12&13 brothers. The R-V1636 form of R1b seen in the Yellow family occurred also at Ekaterinovka Mys, where it was abundant among males, at Progress-2, and at the Eneolithic cemetery at Berezhnovka II on the lower Volga, so seems to have been widespread in the Volga-North Caucasus steppe mating network in the final centuries of the 5^th^ millennium BCE. This is a separate side branch of the R1b that led to the typical Yamnaya form of R1b.

Figure 18:B presents a similar analysis for the Grey family, including the mace chief in II:24 and his sister in II:25, in this case representing one possible family tree among several equally plausible trees. The Grey family had Y-haplogroup Q1a2b (Q-YP1669), a patriline with northern forest-zone and Siberian connections, and three maternal mtDNA haplogroups, including U2e, linked above with an isotopically distinct riverine catchment. None of their haplogroups, maternal or paternal, were shared with the Yellow family. Like the Yellows, the Grey patriline buried at Khvalynsk was divided into two primary avuncular branches. But the Greys also included a male related only through his mother. Grey males II:34 and II:1 were 3^rd^-degree relatives, probably cousins whose mothers were sisters (U5a1i), sharing only maternal relatives in a cemetery dominated by paternal relations (Table 6). Also, II:1’s mother had married an R1a (R-M459) husband, making II:1 the only R1a (R-M459) male at Khvalynsk II. Male II:1 was included in the Grey family through his relationship with his mother’s sister’s son, hinting at the continuing importance of maternal marriage links in this paternally dominated society. Additionally, a male unrelated to anyone was included in grave II:26, with Y-haplogroup J1 (J-CTS1026) (Table 6). Some males at Khvalynsk II were associated with but not genetically related to the others.

As was noted above, the Grey family graves seem to have been arranged around the pre-existing east-west row of Yellow graves, with the Grey mace chief in II:24 located near the copper-rich II:12 male. The Grey family is modeled as present at Khvalynsk over three generations. If the Yellow family was present first, and some overlap is permitted between the two families, then the Yellow and Grey family graves could fit within five generations, or 140 years at 28 years/gen. With no overlap eight generations (224 years) would be required. The two radiocarbon dates on ruminant animal bone from Khvalynsk I and II overlapped in a single century 4450–4350 BCE, supporting a short span of time.

The two dominant patrilines at Khvalynsk II had distinct histories and fates. The Q1a Y-haplogroup is also found at the cemetery of Murzikha II, located 400km north of Khvalynsk in the forests of the Volga-Kama region, and chronologically contemporary with Khvalynsk or slightly later (4400–4100 BCE). Most men at Murzikha II were Q1a, but from a different lineage (Q1a1) than the Grey family at Khvalynsk (Q1a2). A migrant from the steppes buried in Hungary at Csongrad-Kettëshalom Bastanya, contemporary with Khvalynsk, also had Y-haplogroup Q1a2, like the Grey family, and autosomal DNA similar to Khvalynsk. This steppe male was part of a diaspora of steppe males into the Danube valley that occurred about 4400–4200 BCE. The Q1a patriline was then mobile and wide-ranging, and at Khvalynsk II was accorded the richest grave at the cemetery. However, most of the men at Khvalynsk II, and all the Yellow family, were R1b of the R-L754 > R-L389 > R-V1636 lineage. A millennium later, when the Yamnaya culture appeared, the Q1a Y-haplogroup would be eliminated from steppe patrilines and a different branch of the R1b family, R-Z2103, would become dominant.

The society that created Khvalynsk II was organized patrilineally, although at least one maternal cousin was included. The dominant male lineage seems to have shifted from R1b to Q1a. The females were from genetically distinct and unrelated families, except for one sister buried with her brother (25&24). The male-centered ancestries at Khvalynsk II suggest a virilocal kinship system, and the absence of their mothers, daughters, and sisters might indicate that this was a burial place for a multi-generational male sodality, with some unrelated females (wives?) and children.

### Cranio-facial groups and genetic mating networks in the steppes

The cranio-facial types of Khvalynsk and neighboring Eneolithic sites were studied by A. A. Khokhlov in Samara as part of a quantitative metric analysis of 549 skulls from the Volga-Ural region (Khokhlov 2010, 2017). Cranio-facial metrics showed that the Khvalynsk population was an admixture of two major components, one (robust, broad-faced) derived from the northern forest zone and the other (more gracile, narrow-faced) from the southern steppes, a conclusion borne out by aDNA data that came to the same conclusion (see below). Khokhlov further divided each major regional type into two sub-types, so two northern sub-types (Lapp-like and Uralic) and two southern (perhaps lower Don and Caucasus steppe). Khokhlov was uncertain about the exact metric source of the southern component at Khvalynsk, which is also true of the geneticists’ uncertainty about the exact source of the southern genetic component (CHG). His metrics also identified the cranio-facial similarities between most of the first-order relatives discussed above: 12 & 13 brothers, noted as very similar by Khokhlov (2010: 431); 29 & 30 father-son (Khokhlov 2010: 431); 24 & 25 brother-sister (Khokhlov 2010: 431); and 18 & 33 brothers, noted as similar in Khokhlov (2010: 429). Also Khokhlov felt that the Khvalynsk II burial plot was designated for the burial of some special group of males who died young, compared to the males in Khvalynsk I. In many ways, cranio-facial metrics, traditional demographic research, and aDNA findings confirmed each other at Khvalynsk.

The Khvalynsk population was genetically admixed between northern and southern ancestry types, in general agreement with Khokhlov’s interpretation based on cranio-facial data. The northern type, Eastern Hunter-Gatherers (EHG), evolved in northern Eurasia; and the southern type, designated Caucasus Hunter-Gatherers (CHG), was defined initially by Mesolithic and Early Neolithic inhabitants of Georgia and western Iran (Mathieson et al. 2018; Wang et al. 2019; Anthony 2019). Both the EHG and CHG labels were first applied to hunter-gatherers, but afterwards were extended to genetically similar individuals regardless of economy. Wang et al. (2019:3) recognized that EHG & CHG ancestry like Khvalynsk was shared by Eneolithic individuals at Progress-2 (Figure 16) and Vonyuchka-1 (also known as Konstantinovskii-1) in the North Caucasus steppes. They are dated 4336–4173 calBCE (5397±28BP/MAMS-110563); and 4233–4047 calBCE (5304±25BP/MAMS-11210).

We do not know the proximate source of the CHG population that mixed with EHG to create the typical Khvalynsk/Progress-2 pattern of genetic ancestry. But it must have separated from other CHG populations in the Caucasus and western Iran before about 6500–6000 BCE, because after this date (Skourtanioti et al. 2020: 1164) the CHG populations in the Caucasus and western Iran became admixed with Anatolian Farmer (AF) ancestry. By 4700 BCE, when the first farmers migrated from Georgia across the western North Caucasus Mountains and occupied sites on the north side of the North Caucasus ridge such as Meshoko and Svobodnoe, they had up to 50% AF ancestry (Wang et al. 2019). The Progress-2/Khvalynsk steppe people had no AF ancestry, so they did not exchange mates with Meshoko farmers, even if archaeology shows that they did exchange material valuables (see Copper section above).

In the aDNA literature, “steppe ancestry” is a phrase used since Allentoft et al. (2015) and Haak et al. (2015) to refer to the typical Yamnaya pattern of genetic ancestry. The principal components of steppe ancestry were EHG & CHG, each in robust proportions, like Khvalynsk, although often with more CHG than in the Khvalynsk/Progress-2 population, with an added component of Anatolian Farmer (AF) ancestry (5–15%) that was absent from the Khvalynsk/Progress-2 populations (Mathieson et al. 2018: Figure 2; Narasimhan et al. 2019: S.I. 234; Wang et al. 2019: 7). Also, the Khvalynsk/Progress-2 mating network has not yet yielded the Y-haplogroup mutations that were directly ancestral to the typical Yamnaya form of R1b (R-Z2103). The R-V1636 form of R1b, found in males at Khvalynsk, Ekaterinovka Mys, Berezhnovka II, and Progress-2, identifies a branch that split from the Yamnaya branch defined by R-P297 > R-M269 > R-L23 > R-Z2103 (yfull.com). This entire branch is absent from the sampled Eneolithic males from the steppes, appearing for the first time in Yamnaya males. The evolution of Yamnaya Y-haplogroup ancestry occurred in a still-unsampled Eneolithic population.

The Eneolithic populations around the Dnieper Rapids (Telegin and Potekhina 1987) were even more different from Yamnaya. All those sampled were admixtures of EHG (primarily) and Western European Hunter-Gatherers (WHG) similar to the Iron Gates Mesolithic populations (Mathieson et al. 2018). Among 30 published individuals from three Neolithic and Eneolithic cemeteries (Dereivka-1, Vil’nyaka, and Vovnigi) in the Dnieper River valley dated 5200–4400 BC, assigned to the Dnieper-Donets culture, there were a few individuals with minor (<10%) CHG ancestry, but most had none (Mathieson et al. 2018: 198, Fig.2). The Khvalynsk/Progress-2 populations had substantial CHG ancestry but no WHG ancestry, ubiquitous in Dnieper-valley populations. This indicates that the Dnieper-Donets mating network did not extend eastward to the Volga, nor westward to the Criş and early Tripol’ye farmers, whose ancestry was typical of European farmers (AF or EEF) (Schmidt et al 2020). The Dnieper-Donets people seem to have been an endogamous population focused on the rich resources of the Dnieper Rapids. Their substantial WHG ancestry, nearly absent in Yamnaya individuals, rules them out from being a major source for the Yamnaya.

The Sredni Stog culture succeeded and replaced the Dnieper-Donets culture in the strategic Dnieper Rapids and throughout the steppes of Ukraine beginning around 4500–4300 BCE and ending in the late fourth millennium BCE with the appearance of Yamnaya. Unpublished Sredni Stog male genomes exhibit admixture ‘cocktails’ with the same basic elements as Yamnaya (EHG & CHG & AF). The CHG & EHG component was like Khvalynsk/Progress-2, suggesting an eastern origin for at least part of the Sredni Stog population, and the AF component could have come from either the early Maikop or Tripol’ye populations. Sredni Stog introduced into the Ukrainian steppes new funeral customs (the Khvalynsk or ‘Yamnaya’ position), ceramic types (shell-tempered like Khvalynsk), and economies (large numbers of horse bones) that had appeared earlier on the Volga. Sredni Stog has for decades been recognized (Telegin et al. 2001) as an Eneolithic ancestor of Yamnaya influenced by late Khvalynsk, early Maikop, and the Tripol’ye and Varna cultures. But neither R1b Z-2108 nor its immediate ancestral forms are found among sampled Sredni Stog males, most of whom belonged to the R1a or I2a haplogroups, unlike Volga males. The sampled Sredni Stog populations included individuals who autosomally resembled Yamnaya a millennium before the Yamnaya culture appeared. But within that population the Yamnaya Y-haplogroup patriline evolved in a region that has not been sampled.

## Conclusion: Khvalynsk as a coalescent culture

Khvalynsk was an exceptionally large cemetery of the mid-to-late-fifth millennium (4500–4300 BCE from dates on terrestrial animal bones), used by a heterogeneous, admixed population. Cranio-facial measurements and genetic ancestry indicate mixture between northern (forest-zone, EHG) and southern (lower Don-Caucasus, CHG) population components (Khokhlov 2010; 2017). The admixed Khvalynsk population exhibited significant genetic and family diversity. It seems to have been a central place for the ritual integration and unification of a population that normally lived dispersed up and down the Volga in isotopically different catchments. At least one female buried at Khlopkov Bugor, 130 km south on the Volga, was a 2^nd^-degree relative (grandmother or great-aunt) of a Yellow-family male buried at Khvalynsk, but the cemetery at Khvalynsk was ten times larger than at Khlopkov Bugor.

The exclusive use of domesticated cattle, sheep-goats, and horses for funeral sacrifices at Khvalynsk indicates the acceptance of a new set of religious ideas about the desires of the gods and ancestors, who now could be satisfied only by the sacrifice of domesticated animals (horses included). An early phase in the evolution of this cult might be indicated by the few sheep, goat, and horse sacrifices at the older cemeteries at Ekaterinovka Mys and the related site of Sy’ezzhe about 4700–4500 BCE.

About 4500 BCE new long-distance exchange systems connected Khvalynsk with the Varna-era Balkans. Imported copper metal began to flow into the social and political world of steppe societies. These two new imports, copper ornaments and domesticated animals, gave ambitious families at Khvalynsk, ‘aggrandizers’ in Hayden’s terms (Hayden 2009, 2012), two different kinds of status enhancers that could be used to build alliances in a political context that featured war and human trophy-taking. Imported copper was consumed individually, so enhanced individual status; while locally-raised feast animals were consumed communally, so enhanced group solidarity and the generosity of the hosts. At death, people who wore copper ornaments did not normally receive animal sacrifices, and people who received animal sacrifices in the grave did not normally wear copper ornaments, signaling the presence of distinct social segments marked by the two new status enhancers. Polished stone maces were used at Khvalynsk to identify the only adult males who belonged to both segments (copper-users and sacrifice-receivers) simultaneously, arguably to represent the union of both. These mace-chiefs seem to signal the emergence of hierarchy during the Eneolithic, but their maces also symbolized alliance and the agreement of at least two social segments to accept one adult male as their joint representative.

Khvalynsk can be regarded as ‘coalescent culture’, a culture resulting from the integration of cultural components that originally were geographically (north-south), genetically (EHG-CHG), and culturally distinct. Coalescence is different from the concept of hybridity developed in post-colonial studies (Stockhammer 2012), in that coalescence focuses on hybrid communities and bridge-building institutions rather than on individual agency and alterity. The coalescent community model, originally developed to characterize multi-ethnic indigenous societies in the post-Contact U.S. southeast (Kowalewski 2006), was revised to provide an explanatory framework for the dramatic migrations and community reorganizations of the late thirteenth and fourteenth centuries in the U.S. southwest (Clark and Reed 2011: 258; Clark et al. 2018) and the League of the Iroquois (Birch and Hart 2018:26). In the American examples, coalescent communities represented the initial phase of cultural integration during a period of population movement, when cultural assimilation and hybridization processes were incomplete, under political conditions where all forms of extra-familial authority were relatively weak. Pre-existing ethnic and tribal identities as well as indigenous political authority were sustained through metaphors of kinship, common origin, or co-residence that were deeply ingrained and resistant to change even after periods of conflict, chaos, and reorganization. Coalescent communities overcame these deeply ingrained identities through the creation of new meta-identities that functioned beside the older, more limited ones. These meta-identities were founded upon new religious beliefs and rituals such as the Kachina Cult that provided new institutions and sodalities that were accorded a level of prestige equal to those of family or clan (Clark and Reed 2011; Clark et al. 2018). Similarly, at Khvalynsk we witness the appearance of new funeral rituals in which domesticated mammals were the mandatory medium for the ceremonies of a social minority that apparently distributed funeral feasts to hundreds of mourners. This new set of rituals and feasts could have sustained a regional meta-identity that overcame divisions based on local kinship and co-residence. Khvalynsk II might even have been a burial place for a new multi-generational male sodality, an example of a bridging institution.

As was mentioned above (see Mace Holders), the League of the Iroquois shared many features of a coalescent community but was understood by those inside the league as the product of a religious awakening inspired by a culture hero who introduced new, integrative, bridge-building rituals at a time of debilitating warfare and sorcery. The most important ritual, the Condolence Ceremony, was conducted when one of the five (originally) League chiefs died. This was perhaps paralleled in the funerals of the mace holders at Khvalynsk. The League also functioned partly to facilitate and sustain trade between previously hostile communities after intensely desired European trade goods were introduced (Starna 2008; Birch and Hart 2018). The Balkan copper trade could have stimulated integrative institutions in the same way.

The new religious ideas and rituals at Khvalynsk can be seen as elements in the creation of a new meta-identity that unified the previously disparate populations of the Volga-Don-Caucasus steppes. Domesticated animal sacrifices continued into the Yamnaya period, again among a minority, since only about 15% of Yamnaya graves had animal sacrifices (Shilov 1985:25). If Yamnaya is accepted as the material correlate of late Proto-Indo-European languages, then the new religion indicated at Khvalynsk was an important ancestor of Indo-European religious ideas. The first Indo-European priest-figure archetype sacrificed a divine cow from which the world was made, and the Indo-European warrior-hero archetype defeated a serpent who had imprisoned divine cows (or flowing water, in some versions) (Watkins 1995). Domesticated animal sacrifice was at the root of Indo-European religious ideas. Other integrative institutions probably were added to these rituals as the Eneolithic steppe cultures passed through the coalescent phase to emerge as the hybrid cultural background for the Yamnaya culture.

## Figures and Tables

**Table 1:** Khvalynsk I and II radiocarbon dates and stable isotopes. Paired human-terrestrial fauna dates in bold. Note that some individuals were sampled more than once. c2 tests in red italics fail to combine (i.e., they are significantly different).

Grave #	Material	Lab number	Age BP	±	calBC (95%)		calBC mean	δ^13^C	δ^15^N	Notes
**Khvalynsk I**										
Skel. 4	Human F	UPI-119	5903	72	4985	4555	4779			
Skel. 13	Human F	UPI-122	4030	60	2865	2350	2583			
Skel. 17	Human F	PSUAMS-2883	5775	25	4703	4547	4627	−22.9	14.6	
Grave 19	*Shell bead*	Ki-2180	7140	150	6367	5725	6017			*χ* ^*2*^ *, df=2, T=9.3(5% 6.0)*
Grave 19	*Shell bead*	Ki-?	6570	150	5754	5215	5505			
Grave 19	*Shell bead*	Ki-?	6600	150	5801	5220	5533			
Skel. 26	Human F	UPI-120	5808	79	4841	4458	4660			
Skel. 30	Human M	PSUAMS-2884	5995	25	4983	4795	4882	−20.8	15.3	
Skel. 62	Human M	UPI-132	6085	193	5471	4549	5003			
Skel. 127	Human M	GrA-26899	5840	40	4796	4553	4700	−20.7	14.5	*χ* ^*2*^ *, df=2, T=21.0(5% 3.8)*
Skel. 127	Human M	PSUAMS-2885	5625	25	4537	4362	4444	−20.6	14.1	
Skel. 147	Human F	PSUAMS-2886	5845	25	4791	4615	4716	−21.6	14.5	
Grave 147	*Sheep-goat bone ring*	GrA-29178	5565	40	4489	4341	4404	−17.9	11.7	*χ* ^*2*^ *, df=2, T=34.7(5% 3.8)*
**Khvalynsk II**										
Skel. 1	Human M	PSUAMS-4032	5760	25	4697	4539	4612	−20.1	15.6	
Skel. 1	Human M	Univ. Bradford						−20.2	14.8	
Skel. 2	Human F	PSUAMS-2902	5975	25	4940	4790	4859	−21.9	15.4	
Skel. 4	Human M	PSUAMS-2903	5965	25	4938	4737	4846	−21.0	15.6	
Skel. 6	Human F	PSUAMS-4250	6085	25	5204	4905	5002	−22.2	14.8	
Skel. 7	Human M	PSUAMS-4148	5900	25	4836	4715	4767	−21.1	15.9	
Skel. 7	Human M	Univ. Bradford						−20.9	15.3	
Skel. 10	Human F	PSUAMS-4149	6150	25	5209	5006	5109	−23.4	14.7	*χ* ^*2*^ *, (humans): df=1, T=15.9(5% 3.8)*
Skel. 10	Human F	OxA-4311	5790	85	4839	4451	4641	−20.3	14.0	
Grave 10	*Cow bone*	GrA-34100	5570	40	4491	4342	4406	−20.0	7.5	*χ*^*2*^ *(paired), df=1, T=151.8(5% 6.0)*
Skel. 12	Human M	AA-12572	5985	85	5207	4681	4885	−21.5		χ^2^, df=1, T=0.1(5% 3.8)
Skel. 12	Human M	PSUAMS-4031	5960	25	4936	4730	4839	−20.8	15.6	
Skel. 12	Human M	Univ. Bradford						−20.4	15.2	
Skel. 13	Human M	PSUAMS-4200	5985	25	4945	4792	4871	−21.7	16.1	
Skel. 17	Human M	PSUAMS-4033	6070	25	5198	4853	4976	−22.3	14.5	
Skel. 17	Human M							−22.2	13.8	
Skel. 18	Human M	OxA-4314	6015	85	5208	4715	4922	−22.4	13.6	χ^2^, df=1, T=1.6(5% 3.8)
Skel. 18	Human M	PSUAMS-2906	6125	20	5209	4958	5079	−21.8	14.2	
Skel. 19	Human F	PSUAMS-4151	6260	25	5311	5084	5250	−23.4	16.2	
Skel. 22	Human M	PSUAMS-4153	5950	25	4929	4726	4826	−21.0	16.3	
Skel. 23	Human M	Univ. Bradford						−20.6	14.2	
Skel. 24	Human M	OxA-4312	5830	85	4900	4460	4686	−20.2	13.8	χ^2^, df=1, T=3.4(5% 3.8)
Skel. 24	Human M	PSUAMS-4154	5995	25	4983	4795	4882	−21.1	16.1	
Skel. 25	Human F	PSUAMS-4162	5730	25	4678	4494	4576	−20.8	16.6	
Skel. 26	Human M	PSUAMS-4163	6100	25	5206	4935	5032	−21.9	16.9	
Skel. 27	Human M	PSUAMS-4304	6000	25	4987	4797	4888	−21.9	16.3	
Skel. 28	Human M	PSUAMS-4545	5820	25	4783	4555	4676	−20.5	15.6	
Skel. 29	Human M	PSUAMS-4150	5840	25	4789	4613	4708	−21.1	16.6	
Skel. 30	Human M	AA-12571	6200	85	5359	4935	5140	−20.5		removed from model *χ*^*2*^*, df=1, T=6.0(5% 3.8)*
Skel. 30	Human M	PSUAMS-4223	5985	25	4945	4792	4871	−21.5	15.7	
Skel. 31	Human M	PSUAMS-4305	5930	25	4889	4722	4799	−21.6	15.5	
Skel. 32	Human F	Univ. Bradford						−21.1	15.4	
Skel. 33	Human M	PSUAMS-4164	5640	25	4540	4369	4466	−21.3	15.3	
Skel. 34	Human M	OxA-4313	5920	80	5001	4555	4802			removed from model *χ*^*2*^*, df=1 T=4.3(5% 3.8*
Skel. 34	Human M	PSUAMS-4306	6095	25	5206	4909	5022	−22.6	15.5	
Skel. 35	Human M	PSUAMS-4155	6150	25	5209	5006	5109	−22.9	15.1	χ^2^, df=1, T=1.7(5% 3.8)
Skel. 35	Human M	OxA-4310	6040	80	5209	4729	4953			
Skel. 38	Human M	PSUAMS-4156	5755	25	4695	4508	4607	−20.9	16.2	

**Table 2:** Fauna in graves and sacrificial deposits at Khvalynsk I and II, MNI only. Compiled from the sources listed in the first sentence of this section.

	Khvalynsk I sacrificial deposits	Khv I graves	Khvalynsk II sacrificial deposits	Khv II graves	Total MNI	Percent
Cattle	10	13	2	4	29	19.2%
Sheep-goat	29	44	4	29	106	70.2%
Horse	4	7	2	3	16	10.6%
Total	43	64	8	36	151 MNI	

**Table 3:** All graves, with age and sex, containing animal sacrifices (by species), copper objects, and stone maces at Khvalynsk I and II. Does not include above-grave sacrificial deposits. Comments such as “burned” are from Agapov et al. 1990 text and Table 1.

Skel. #	Sex age	Sheep-goat	Cattle	Horse	Copper	Mace
I: 7	f 50–70	X				
I: 11	i 5–7	X				
I: 19	m 50–60				2 rings	
I: 30	m 50–60				2 rings 2 beads	
I: 49	m 40–50				1 ring	
I: 57 55 56	m 40–50 m 40–50 i 13–14	X	X burned		2 rings	1 cruciform mace
I: 64	f 50–60			X		
I: 67	m 50–60				1 ring	
I: 68	f 50–60	5 talus bones from 4 individuals				
I: 71	f 40–50				2 linked rings	
I: 72	f 40–50				2 linked rings	
I: 74	f 15–20				1 ring	
I: 90	i 10–14				1 ring	
I: 93	i 6–10		X burned			
I: 97	f 60–70	from 5 individuals				
I: 100	i 4–6	back leg of a lamb	X			
I: 102	m adult				1 ring	
I: 104	f 25–35				1 ring	broken tip
I: 106	i 4–7		X			
I: 108–110	m adult	X			1 ring	1 eared mace 1cruciform mace
I: 113	m 30–40	X				
I: 115	f 17–25	35 talus from 22 ind				
I: 123	f 25–35				1 ring	
I: 126	m 17	vertebrae 2 individuals				
I: 127	m adult	X		X		
I:129	f 20–25				1 ring 1 spiral	
I: 131	m adult			X		
I: 132	m adult			X		
I: 133	i 10–13				trace	
I: 139	m 30–40	X	X			
I: 140–141	m 45–55 f 30–35	1 skull	?	?		
I: 143–144	m 45–50 f 30–40		skull fragments of 8 individuals	X		
I: 145	m 40–60			X		
I: 155	f 11–15	X				
I: 157	m 50–60			X		
**Skel. #**	**Sex age**	**Sheep-goat**	**Cattle**	**Horse**	**Copper**	**Mace**
II: 1	m 30–35				1 ring 1 bead	
II: 2	f 17–20				trace	
II: 3	i 4–5				1 pendant 1 curved sheet frag	
II: 6	f 20–30	X			2 rings	
II: 10	f 55–65		X			
II:12	m 20–30				293 beads 2 rings 2 pendants	
II: 13	m 25–35				2 rings 1 bead	
II: 14	i 1 yr			X	1 bead	
II: 16	i 1 yr	X				
II: 18	m 45–55				1 ring	
II: 21	m 50–55				melted lump	Antler hammer
II: 24	m 20–25	X			1 spiral pendant 3 melted lumps 8 rings 4 beads	1 eared mace
II: 26	m 17–22				trace	
II: 31	m 20–30				1 ring	
II: 35	m 40–50				1 pendant	
II: 38	M	X	X			

**Table 4.** Ekaterinovka Mys radiocarbon dates. Intcal20

Grave #	Lab	Sampled material	Age BP	calBC (95%)	δ^13^C	δ^15^N
grave 40	PSUAMS 8194	beaver incisor	5750 ± 25	4686–4505	−20.7	6.7
grave 45	PSUAMS 4568	goat tooth	5680±20	4550–4450	Nd	
grave 101	PSUAMS 8195	sheep tooth	6025 ± 40	5028–4798	Nd	
Grave 60	PSUAMS 8218	marmot tooth	5745±30	4689–4517	Nd	
potsherd	SPb-2251	organic residue	5673±120	4795–4267	Nd	

**Table 5.** Binary pairs of relatives at Khvalynsk II with related individuals coded by color.

Degree	Lab ID	Lab ID	Relationship	Family	Grave numbers
**1st degree**	I0122	I6403	Brothers	yellow	12 bro of 13
	I6403	I6406	Father-son	yellow	13 fa of 22
	I6299	I6739	Brothers	green	18 bro of 33
	I6407	I6734	Brother-sister	grey	24bro & 25sis
	I6408	I6737	Father-son	orange	35 fa of 28
	I6741	I6402	Father-son	purple	30 fa of 29
**2**^**nd**^ **degree**	I0122	I6406	Uncle-nephew	yellow	12 pa uncle of 22
	I0122	I6736	“	yellow	12 pa uncle of 27
	I6403	I6736	“	yellow	13 pa uncle of 27
Khlopkov Bugor & Khvalynsk	I6301	I6107	“	yellow	K.B.7 grandma of Khv. II:4
	I0434	I6740	“	grey	17 ma uncle of 34
				grey	17 pa uncle of 23
**3**^**rd**^ **degree**	I6406	I6736	Cousin / great-grandfather	yellow	22 cousin/gr-grafa of 27
	I0122	I6738	“	yellow	12 “ of 31
	I0122	I6109	“	yellow	12 “ of 7
	I0122	I6107	“	yellow	12 “ of 4
	I6403	I6107	“	yellow	13 “ of 4
	I6403	I6109	“	yellow	13 “ of 7
	I6403	I6738	“	yellow	13 “ of 31
	I6107	I6109	“	yellow	4 “ of 7
	I0434	I6734	“	grey	17 “ of 25&24
	I0433	I6740	“	grey	1 “ of 34
	I6741	I6412	“	purple	30 “ of 38

**Table 6.** Y and mtDNA haplogroups at Khvalynsk and the relative at Khlopkov Bugor.

Lab ID	mtDNA haplogroup	Cemetery & grave #		Y-haplogroup v1
I6412	H13a2a	Khvalynsk II, Grave 38	M	R1b-L754
I6403	H2a1	Khvalynsk II Grave 13	M	R1b-L754
I0122	H2a1	Khvalynsk II Grave 12	M	R1b-L754-L389-V1636
I6402	H2a1	Khvalynsk II Grave 29	M	R1b-L754
I6738	R1b1	Khvalynsk II Grave 31	M	R1b-L754
I6106	T2a1b+723+10005	Khvalynsk II Grave 2	F	
I6102	T2a1b	Khvalynsk I Grave 17	F	
I6104	U2e1a1	Khvalynsk I Grave 127	M	R1b-L754-L389
I6407	U2e1b	Khvalynsk II Grave 24	M	Q1-L472-M25-YP1669
I6734	U2e1b	Khvalynsk II Grave 25	F	
I6105	U2e1b+8494+15287	Khvalynsk I Grave 147	F	
I6299	U2e2a1	Khvalynsk II Grave 18	M	Q1-L472-M25-YP1669
I6739	U2e2a1	Khvalynsk II Grave 33	M	Q1-L472-M25-YP1669
I6108	U4a	Khvalynsk II Grave 6	F	
I0426	U4a+6524+9989+12308	Khvalynsk II, Grave 32	F	
I6735	U4a+6524+9989+12308	Khvalynsk II Grave 26	M	J1-CTS1026
I6408	U4a1	Khvalynsk II Grave 35	M	R1b-L754
I11837	U4a1+8155+13158+489+3780+13635	Khvalynsk I Grave 40	M	R1b-L754
I6741	U4b1+293+13834	Khvalynsk II Grave 30	M	R1b-L754
I0434	U4d+16240	Khvalynsk II Grave 17	M	Q1-L472
I6110	U4d+16240	Khvalynsk II Grave 10	F	
I6406	U4d+10692+13708+15544+16093	Khvalynsk II Grave 22	M	R1b-L754
I6109	U5a1+16192	Khvalynsk II Grave 7	M	R1b-L754-L389
I6404	U5a1	Khvalynsk II Grave 19	F	
I6736	U5a1a1	Khvalynsk II Grave 27	M	R1b-L754
I6301	U5a1a1	Khlopkov Bugor, Grave 7	F	
I6737	U5a1a2	Khvalynsk II Grave 28	M	R1b-L754
I6740	U5a1i	Khvalynsk II Grave 34	M	Q1-L472-M25
I0433	U5a1i	Khvalynsk II Grave 1	M	R1a-M459
I6107	U5a2d+146	Khvalynsk II Grave 4	M	R1b-L754-L389
I6405	U5a2d+146	Khvalynsk II Grave 21	M	R1b-L754
I6103	U5a2d+2244+9577+13886+16086	Khvalynsk I Grave 30	M	I2a-L699

## Footnotes

2 Yamnaya culture sites were called Late Neolithic or Eneolithic in the 1950s (Gimbutas 1956: 89–92) but radiocarbon dates, accumulating metal artifacts from Yamnaya graves, and typological links with the late Maikop culture prompted the acceptance of the Yamnaya culture as the beginning of the EBA by the 1980s. N.I. Merpert’s classic synthesis of the Yamnaya culture (Merpert 1974) included the Repin site and Mikhailovka II in the early Yamnaya phase, and this was correlated with the start of the EBA and the ‘Circumpontic Metallurgical Province’ (Chernykh 1992), a chronological terminology that remains dominant today (Trifonov 1996; Shishlina 2008). Proposals to make the EBA begin earlier than Yamnaya (Morgunova 2014, a high EBA chronology) or to make Yamnaya and the EBA begin after the Repin phase (Rassamakin 2013, a low EBA chronology) are debated.

3 The Vonyuchka-1 Eneolithic grave site (kurgan 1, grave 8) was discovered in 2010 in the eastern suburbs of Pyatigorsk. Local archaeologists named it after an adjacent slow stream called ‘the Stinker’. Archaeologists collaborating with Wang et al. (2019) later changed this unfortunate site name to Konstantinovskii-1, after a town northeast of the site (Korenevskii et al. 2019:161). But Wang et al. (2019:3) had already published the whole genome under the name Vonyuchka-1. Here we use ‘Vonyuchka-1 (also known as Konstantinovskii-1)’.

4 Sredni Stog means ‘Middle Stack’ (stack/stog are Indo-European cognates), referring to the central one of three haystack-shaped islands in the Dnieper River where artifacts of this culture were first described in 1927 by A.V. Dobrovol’sky.

5 The Oxford (OxA 4311) date for the human in grave 10 (Table 1) was published by Shishlina et al. (2009: Table 8) and used a bone sample sent from Samara to the State Museum in Moscow in the 1990s. The PSUAMS date (Table 1) was on a petrous bone obtained recently in the anthropology lab in Samara from the skeleton numbered II:10. The lab has moved and been reorganized significantly since the 1990s. The difference between the OxA and PSU dates and stable isotope measurements suggests that they were not from the same individual. We can be reasonably confident that both were from graves at Khvalynsk II, and one of them was from the female in grave 10.
